# Combined effect of nitrogen-doped carbon and NiCo_2_O_4_ for electrochemical water splitting

**DOI:** 10.1038/s41598-024-74031-1

**Published:** 2024-11-06

**Authors:** Laura Kubińska, Mariusz Szkoda, Malgorzata Skorupska, Patrycja Grabowska, Marta Gajewska, Jerzy P. Lukaszewicz, Anna Ilnicka

**Affiliations:** 1 Faculty of Chemistry, Nicolaus Copernicus University in Torun, Gagarina 7, 87-100 Torun, Poland; 2 Faculty of Chemistry, Department of Chemistry and Technology of Functional Materials, Gdańsk University of Technology, Narutowicza 11/12, 80-233 Gdańsk, Poland; 3 Advanced Materials Center, Gdańsk University of Technology, Narutowicza 11/12, 80-233 Gdańsk, Poland; 4 Academic Centre for Materials and Nanotechnology, AGH University of Krakow, Mickiewicza 30, 30-059 Kraków, Poland; 5 Centre for Modern Interdisciplinary Technologies, Nicolaus Copernicus University in Torun, Wilenska 4, 87-100 Torun, Poland

**Keywords:** Hydrogen evolution reaction, Oxygen evolution reaction, Electrocatalyst, Green hydrogen, Hybrid materials, Chemistry, Energy science and technology, Materials science, Nanoscience and technology

## Abstract

Electrocatalytic water splitting for green hydrogen production necessitates effective electrocatalysts. Currently, commercial catalysts are primarily platinum-based. Therefore, finding catalysts with comparable catalytic activity but lower cost is essential. This paper describes spinel-structured catalysts containing nickel cobaltite NiCo_2_O_4_, graphene, and additionally doped with heteroatoms. The structure and elemental composition of the obtained materials were analyzed by research methods such as TEM, SEM-EDX, XRD, XPS, and Raman spectroscopy. The electrochemical measurements showed that hybrid materials containing nickel cobaltite NiCo_2_O_4_ doped with graphene are highly active catalysts in the hydrogen evolution reaction (Tafel slopes = 91 mV dec^−1^, overpotential = 468 mV and onset potential = -339 mV), while in the oxygen evolution reaction (Tafel slopes = 51 mV dec^−1^, overpotential = 1752 mV and onset potential = 370 mV), bare NiCo_2_O_4_ without the addition of carbon has a worse activity (for HER: Tafel slopes = 120 mV dec^−1^, overpotential - does not achieve and onset potential = -404 mV, for OER: Tafel slopes = 54 mV dec^−1^, overpotential = 1796 mV and onset potential = 410 mV). In terms of stability, comparable results were obtained for each synthesized compound for both the HER and OER reactions.

**Supplementary Information:**

The online version contains supplementary material available at 10.1038/s41598-024-74031-1.

## Introduction

The realization of large-scale H_2_ production from water splitting requires the use of electrocatalysts. Despite many advances in this field, several challenges remain. Great efforts have been made to develop electrocatalysts that do not contain noble metals, which will more favorably affect the electrolysis process. The catalyst during the water electrolysis process should exhibit high catalytic activity, and good stability, and it is desirable that the cost of obtaining the catalyst should be acceptable not only on a laboratory scale but also on an industrial scale. Much research has been devoted to developing efficient catalysts for both the hydrogen evolution reaction (HER) and the oxygen evolution reaction (OER). To date, the HER reaction, as well as the OER reaction, are generally catalyzed by precious metal-based catalysts such as Pt, Ru, and Ir, and their oxides. However, the low reserve and high cost of precious metals greatly limit their practical application. Hence, it is very important to develop alternative catalysts that show excellent catalytic activity and stability but have a low price. Therefore, it is highly desirable to develop catalysts for efficient water splitting that will simultaneously work well as bi-functional materials^1–3^. The divergence of reaction conditions at the stage of developing catalysts for HER and OER reactions results in low efficiency of full water splitting. Therefore, the development of non-precious bi-functional electrocatalysts for both HER and OER in the same electrolyte is being pursued to achieve overall water electrolysis efficiency due to the simplification of electrolyzer configuration and reduction of total cost^4–6^. Due to the nature of the four-electron pathway, the OER overpotential is usually much higher than the HER potential, so the overall efficiency of the water cleavage process in alkaline media is more efficient than in acidic media. In addition, most OER catalysts are unstable in strongly acidic electrolytes^7–9^.

In the last few years, researchers have discovered the potential of bimetallic oxides exhibiting synergistic properties as bifunctional catalysts for hydrogen and oxygen evolution reactions^10,11^. These compounds may consist of spinel structures such as ACoO_x_^12,13^, AMoO_x_^14,15^ AFeO_x_^16,17^ or AMoP^18–20^, where A is Co, Zn, Fe, Ni, Cu, or perovskite structures with the general formula ABO_3_, where A is rare earth metal or alkali earth metal with a coordination number of twelve, B is transition metal with a coordination number of six^11^. Despite low conductivity of NiCo_2_O_4_, limited number of active sites, or proclivity towards agglomeration, it exhibits excellent practical properties such as abundant elemental resources, environmentally friendly properties, and industrial-scale applicability^21–23^. Consequently, an array of strategies is increasingly being employed to enhance the electrocatalytic efficacy of the material, often predicated on morphology control, doping techniques, or the integration of the spinel structure with heteroatom-doped carbon materials^21,24,25^. To enhance efficiency, carbon materials are tailored to serve as nanostructured charge carriers, providing a large specific surface area, thereby enhancing accessibility to active sites for the electrolyte and improving material stability^25,26^. This strategy primarily involves the use of carbon nanotubes, graphene, or carbon fibers^27–29^. This action improves the conducting properties and durability of composite materials, thereby preventing the agglomeration of spinel nanoparticles^30,31^. Carbon materials are mainly used due to their large specific surface area, excellent electrical conductivity, and potential for a three-dimensional structure, rendering them an excellent substrate for spinel materials^31,32^. Carbon materials enable an increase in density or the number of active sites through the dispersion of metallic or bimetallic structures^33^. Composite materials consisting of bimetallic structures and carbon effectively reduce electrical resistance and prevent uncontrolled agglomeration of spinel structures^34,35^. Additionally, the use of carbon doped with heteroatoms such as nitrogen changes electrical conductivity and causes modification of the electronic structure of carbon due to the presence of nitrogen atoms in the structure, inducing a positive charge on the adjacent carbon, thereby promoting rapid adsorption of intermediate products^28,35^. Therefore, combining metallic structures with carbon materials additionally enriched with heteroatoms such as nitrogen becomes a favorable solution due to the high affinity of the nitrogen atom to the metal, resulting in the stimulation of interfacial electron transfer^25,28^. There are still many issues that need to be addressed in order to obtain effective and efficient bifunctional catalysts. Therefore, in this paper, we present one of the solutions for obtaining stable spinel structures supported by nitrogen-doped carbon materials.

Recent studies have demonstrated that structural modifications and compositional tuning can significantly influence the electrocatalytic performance of NiCo_2_O_4_. For instance, Li et al. (2019)^36^ reported the synthesis of NiCo_2_O_4_ nanoflowers supported on graphene nanosheets, achieving notable OER activity. Tong et al. (2016)^37^ showcased the benefits of mesoporous NiCo_2_O_4_ nanoplates on graphene foam for the oxygen reduction reaction (ORR). Additionally, Debata et al. (2018)^38^ synthesized graphene-supported NiCo_2_O_4_ coral-like nanostructures, which showed promising results for overall water splitting. Despite these advancements, the exploration of doping strategies, particularly nitrogen doping, in conjunction with NiCo_2_O_4_ remains underdeveloped. Nitrogen doping is known to alter the electronic structure of catalysts and introduce additional active sites, thereby enhancing catalytic activity and stability. Our study addresses this gap by investigating the combined effect of nitrogen-doped functional groups and NiCo_2_O_4_ on the electrochemical performance for HER and OER. By synergistically integrating nitrogen-doped functional groups with NiCo_2_O_4_, we aim to develop a highly efficient and durable electrocatalyst for comprehensive water splitting applications.

The aim of the present study was to develop and synthesize nickel cobaltite NiCo_2_O_4_-containing hybrid materials in such a way as to improve the catalyst performance in the water splitting reaction by adding of some carbon species like doped graphene. The synthesized materials were characterized by physical and chemical analyses such as SEM, SEM-EDX, Raman spectroscopy, and X-ray diffraction analysis. In order to check the catalytic performance, electrochemical tests were carried out on the materials obtained, tests were conducted for hydrogen evolution reaction and oxygen evolution reaction. The specific objectives in this work include proposing a method for obtaining a hybrid material containing nickel cobaltite and carbon in its composition, doping the hybrid material with nitrogen heteroatoms or simultaneous introduction of nitrogen and sulfur heteroatoms. It was very important to check the effect of the addition of heteroatoms and graphene on the electrochemical properties of the obtained catalysts and to compare the catalytic properties of the obtained compounds with commercial catalysts.

## Results and discussion

### Materials characterization

**Fig. 1:**
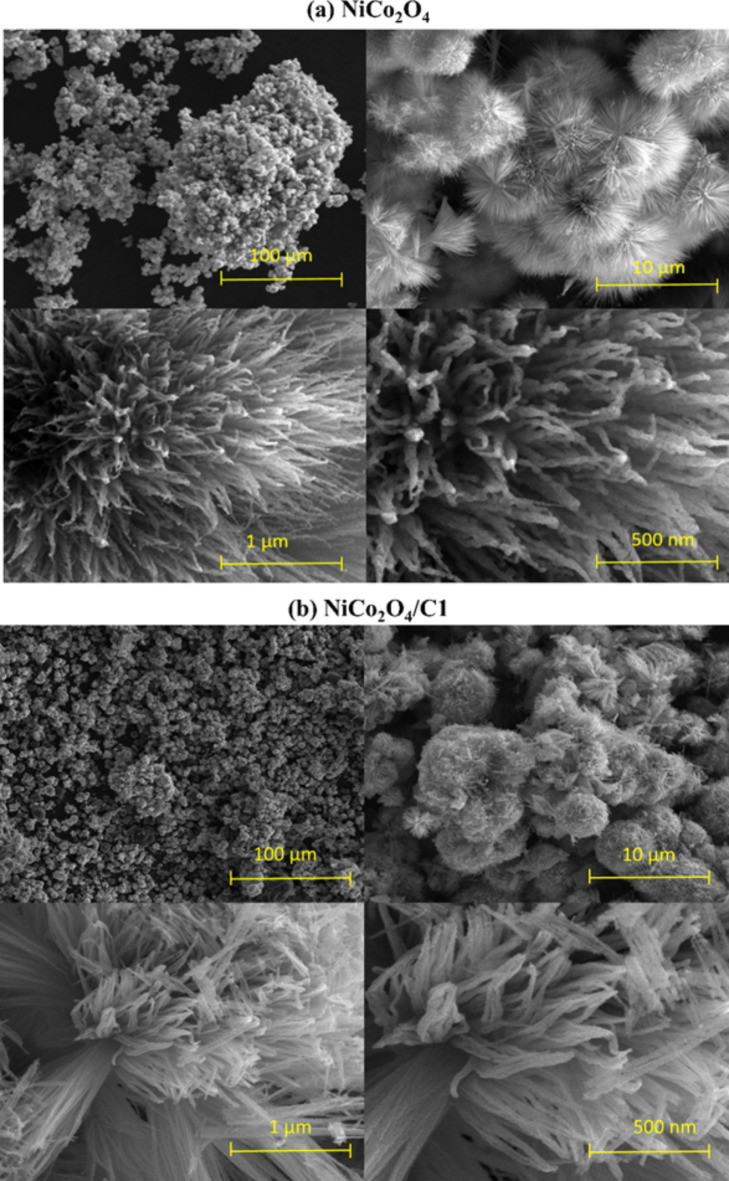
SEM images of samples (**a**) NiCo_2_O_4_ and (**b**) NiCo_2_O_4_/C1 with different magnifications.

Figure 1 shows images obtained with a scanning electron microscope. The NiCo_2_O_4_ catalyst is characterized by a flower-like structure with a spherical shape (Fig. 1a). For the NiCo_2_O_4_ sample at magnification with a scale of 10 μm, the structure resembles a flowering dandelion while at higher magnification values the structure resembles dense rods. Figure 1b and Figure S1 show images for NiCo_2_O_4_/C1 and NiCo_2_O_4_/C2 samples, respectively. These samples have been enriched with carbon material, so their structure has changed. The carbon material they contained (GF750 graphene) caused the partial breakdown of the spherical structures visible for NiCo_2_O_4_ into structures resembling a dense network. Surface morphology of NiCo_2_O_4_/C3 (Figure S2) resembles densely arranged spheres. The structure of these spheres at higher magnification is made up of tightly stacked rods of a similar length but shorter than for samples with carbon C1 and C2. For heteroatoms-doped hybrid materials of NiCo_2_O_4_ and C3 carbon depending on their doping with N and S of NiCo_2_O_4_/C3/N/S (Figure S3) and with only S for NiCo_2_O_4_/C3/S (Figure S4) structure surface structure significant changes is observed. In case of dual-doped NiCo_2_O_4_/C3/N/S sample rods are longer than for pristine NiCo_2_O_4_/C3 hybrid. The structure of single-doped NiCo_2_O_4_/C3/S hybrid is different than other samples and structure was formed in the shape of disks. The observed differences in morphology between the sample containing sulfur and the other samples may be attributed to several factors associated with the introduction of sulfur during the synthesis process. Sulfur has a high affinity for certain elements, and its introduction during the synthesis could lead to chemical interactions with the nickel cobaltite catalyst and carbon materials. These interactions may influence the nucleation and growth processes, resulting in the formation of unique structures. Moreover, sulfur can influence the thermal behavior of the synthesis process. Changes in the annealing conditions or the reaction kinetics due to the presence of sulfur may impact the final morphology of the material.

**Fig. 2:**
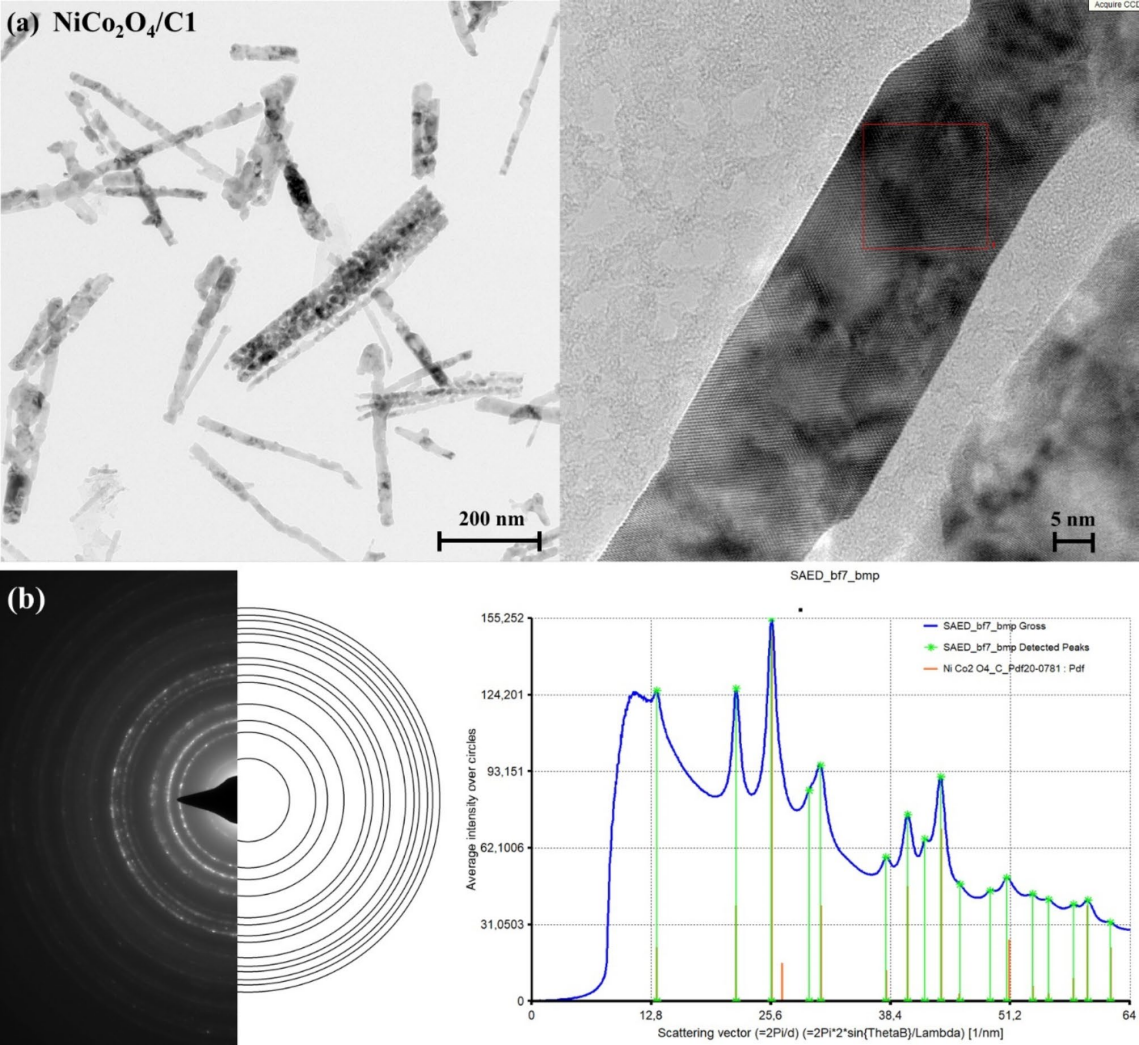
(**a**) TEM images with different magnifications, (**b**) SAED pattern of NiCo_2_O_4_/C1 and intensity profile taken from the SAED pattern.

Transmission electron microscopy (TEM) and selected area electron diffraction (SAED) revealed that the nanoparticles were crystalline. Figure 2a shows TEM images of the NiCo_2_O_4_/C1 sample, indicating a uniform morphology. Similarly, the NiCo_2_O_4_/C3/S sample (Figure S5a), containing nickel cobaltite NiCo_2_O_4_, and the NiCo_2_O_4_/C3/N/S sample (Figure S6a) exhibit particles of similar size. The SAED patterns for NiCo_2_O_4_/C1, NiCo_2_O_4_/C3/S, and NiCo_2_O_4_/C3/N/S were identified using the International Centre for Diffraction Data (ICDD) database. The electron diffraction patterns (Fig. 2b, Figure S5b, Figure S6b) confirm the presence of nickel cobaltite NiCo_2_O_4_ in all analyzed samples.

**Fig. 3:**
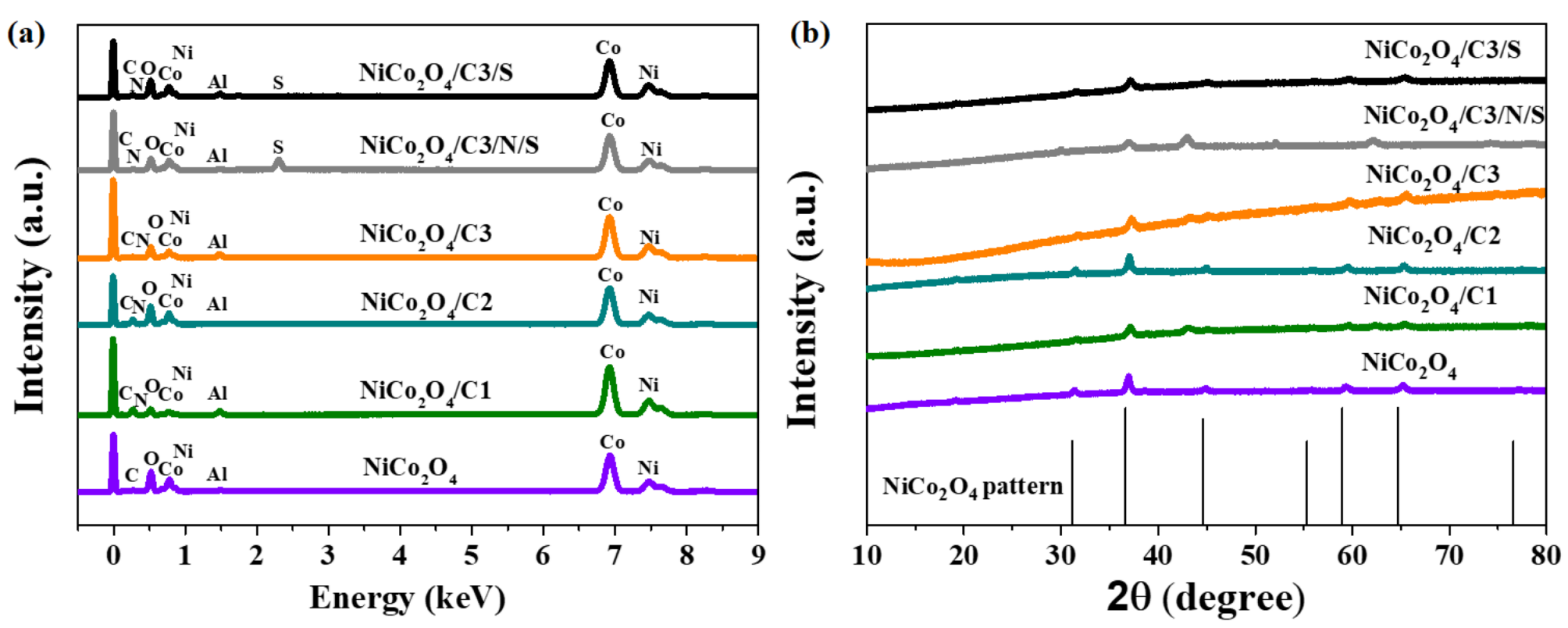
(**a**) EDX spectra of the surfaces confirming their elemental composition; (**b**) X-ray diffraction patterns for NiCo_2_O_4_ and their hybrids with carbon sample.

In order to confirm the elemental composition of the samples obtained, spectra were performed using an EDX detector. Thanks to the performed analysis of the elemental composition of the surface of the samples (Fig. 3a), two signals indicating the presence of cobalt can be observed, moreover, in each material the signal coming from cobalt is quite high, which confirms the dominant presence of this element for all analyzed samples. The intensity of the bands is proportional to the amount of the element in the sample. A smaller signal is also seen from nickel, several times weaker than that from cobalt. On the EDX profiles also are identified bands comes from carbon and oxygen, where oxygen content is assigned to presence in NiCo_2_O_4_ spinel structure in each of the samples obtained. Surprisingly, carbon band is observed in all samples not only in hybrid materials.

The detailed elemental composition of the tested catalysts, obtained using the EDX detector, is presented in Table S1 in the Supplementary Information. The elements C and Al originate from the substrate on which the sample is placed. Additionally, a very small amount of carbon in the sample may be attributed to residues from urea decomposition. Due to high background counts in SEM-EDX, artificial carbon and aluminum peaks are visible in the NiCo_2_O_4_ sample, even though these elements are not present in the specimen. Generally, EDX analysis is not ideal for examining the carbon content of samples. The actual values (% wt.) for NiCo_2_O_4_/C hybrid samples, which contain additional carbon (C1, C2, or C3) added during synthesis, will be higher in combustion elemental analysis but only range from 1.86% weight to 6.84% weight in EDX. According to the EDX analysis, approximately 41.89 to 56.79% weight of the elemental composition in the samples is cobalt. The oxygen content ranges between 17.22% weight and 26.86% weight, while the nickel content ranges from 14.60% weight to 18.29% weight. The average nitrogen content for the samples NiCo_2_O_4_/C3, NiCo_2_O_4_/C3/N/S, and NiCo_2_O_4_/C3/S is 2.01% weight, 3.71% weight, and 2.67% weight, respectively. The highest nitrogen and sulfur content were recorded for the NiCo_2_O_4_/C3/N/S sample, with values of 3.71% weight and 5.04% weight, respectively. The addition of thiourea during the synthesis of the NiCo_2_O_4_/C3/N/S sample influences the composition of these elements. Additionally, the inclusion of methylsulfonylmethane during synthesis of NiCo_2_O_4_/C3/N/S sample resulted in a sulfur content of 0.17% weight. The elemental mapping images for NiCo_2_O_4_ (Figure S7a) confirm the presence of uniform distribution of all detected elements. Figures S7, S8, S9, S10, and S11 show maps images of Co, Ni, and O which are uniformly distributed of the all hybrids (NiCo_2_O_4_/C) over the carbon surface. The colors in the images correspond to the intensity of the X-ray signal for each element for the image confirming the presence of nickel, a more intense green color indicates an area concentrating a greater amount of the described element at a given location. Through EDX analysis, it can be seen that the materials with the addition of carbon material show a more uniform dispersion of oxide-derived elements on the surface of the material than in the case of NiCo_2_O_4_ catalyst samples.

X-ray diffractograms for the obtained catalysts have bands originating from the spinel nickel cobaltite NiCo_2_O_4_ pattern (Fig. 3b). The X-ray diffraction pattern of NiCo_2_O_4_ showed a face-centered cubic symmetry of crystalline structure that closely matched the JCPDS card no 20–0781. The bands analyzed for the obtained materials are relatively broad, indicating the presence of smaller crystallites in the structure^39^. The bands corresponding to the crystal planes of the standard indicate that NiCo_2_O_4_ has a polycrystalline spinel structure. The XRD bands identified in all diffractograms are the same and with the same intensity for both pristine NiCo_2_O_4_ and the NiCo_2_O_4_/C hybrids containing carbon, indicating the high purity of the NiCo_2_O_4_ identified in all samples. The absence of additional characteristic bands for NiO and Co_3_O_4_ on the diffractograms confirms the purity of the NiCo_2_O_4_ crystals obtained^40,41^.

**Fig. 4:**
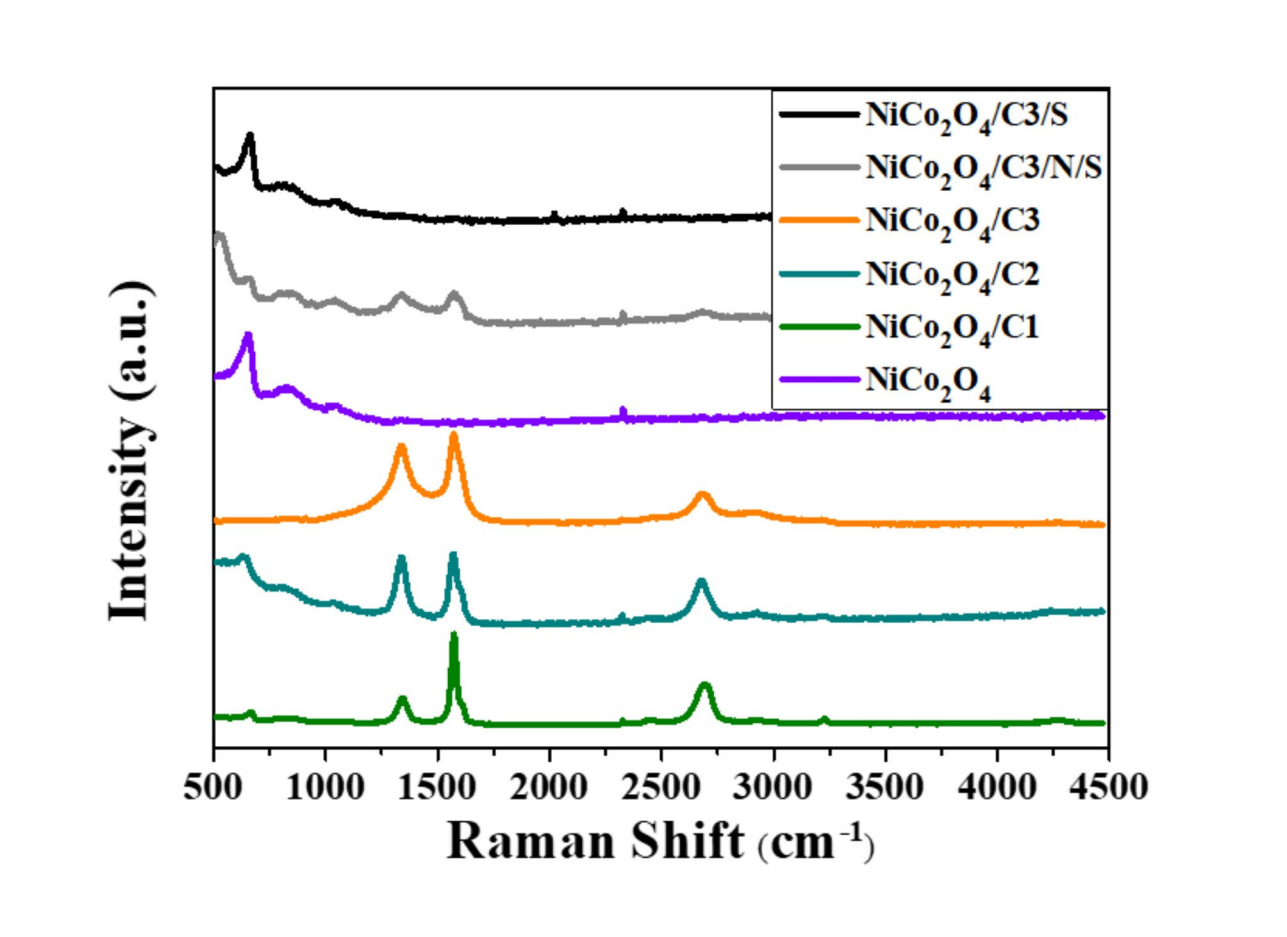
Raman spectra for NiCo_2_O_4_ and their hybrids of NiCo_2_O_4_/C.

Analysis of the Raman spectra makes it possible to determine the structure of the materials and confirm the presence of carbonaceous material. The Raman spectra are shown in Fig. 4. For the NiCo_2_O_4_/C1 and NiCo_2_O_4_/C2 samples, a band was identified at 630 cm^−1^, which is described as A1g and indicates that the particles present in the sample are symmetric. This means that the particle is symmetric at all symmetry operations^42,43^. In addition, three bands can be observed on the Raman spectrum: the D, G, and 2D bands. The D band indicates the presence of free spaces or dislocations in the graphene structure, and the Raman shift of this band is 1355 cm^−1^. The width of the area under the D peak determines the content of amorphous carbon in the sample, while the intensity is an indicator of the presence of defects in the structure. The G band on the spectrum occurs at 1570 cm^−1^, the shape of this band, its width are related to the degree of dispersion of the sample under study, and the intensity of the peak is an indicator of the ordering of the graphene structure. The 2D band is responsible for the presence of sp^2^-configuration bonds for graphene and occurs at a value of 2680 cm^−1^. Based on the analysis of the spectra of all graphene-containing samples, it can be concluded that the 2D band is about twice less intense than the G band, and a smaller than 1 value of the ratio of 2D/G band intensities indicates the presence of single graphene layers that overlap each other, forming multilayer graphene. In addition, for pure graphite, the Raman shift value for the G band is 1583 cm^−1^, and for the materials analyzed, the band has shifted to 1570 cm^−1^, which is due to the presence of heteroatoms in the carbon structure^43,44^. Only in case of NiCo_2_O_4_/C3/S sample on Raman spectrum bands comes from graphene are not visible an spectrum. The structural dissimilarity of this sample was also observed on SEM images as we described in an earlier section of this publication. For NiCo_2_O_4_/C3/S sample observed differences in morphology are attributed to the introduction of sulfur during the synthesis process and sulfur have impact for the final surface morphology of the material.

**Fig. 5:**
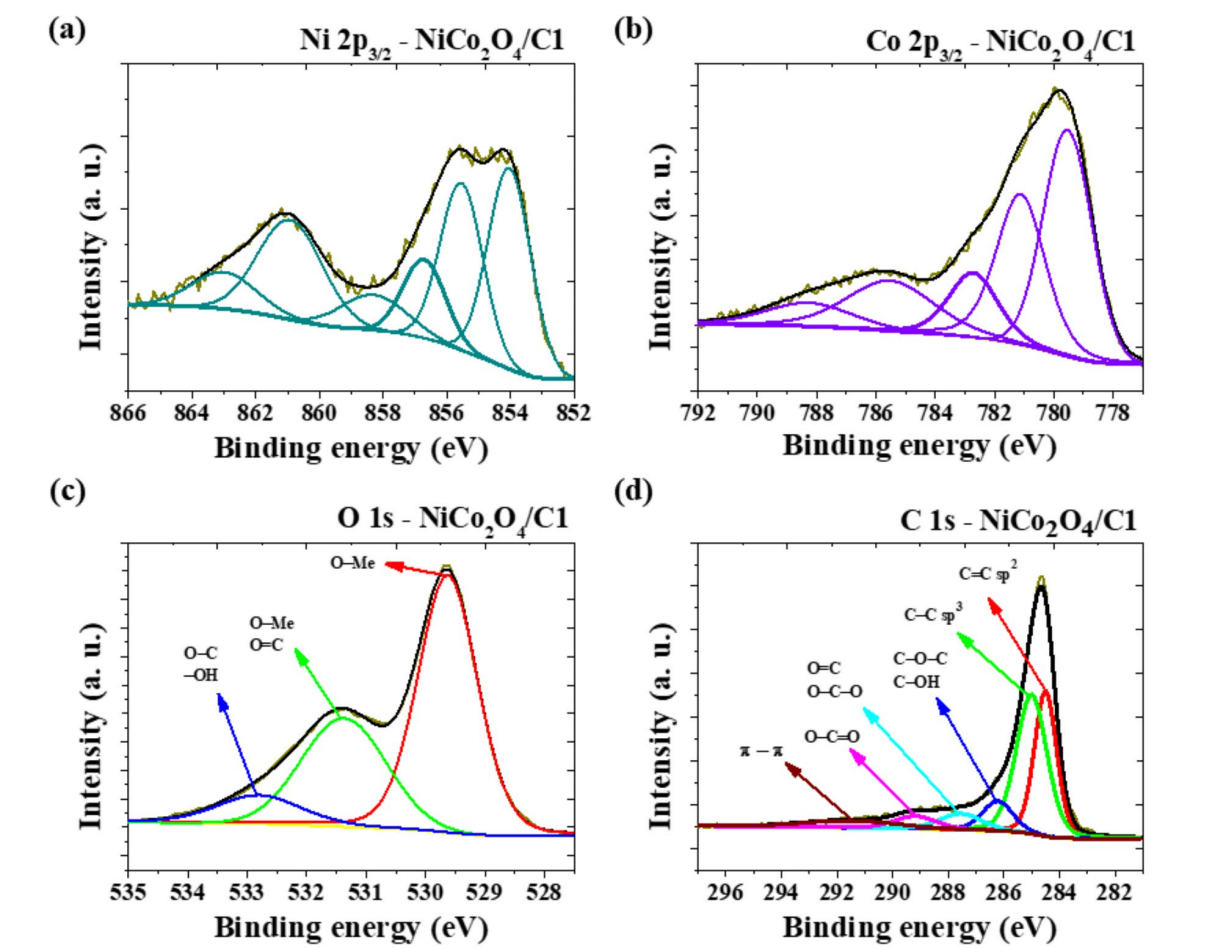
High-resolution XPS spectra of (**a**) Ni 2p_3/2_, (**b**) Co 2p_3/2_, (**c**) O 1s, (**d**) C 1s for NiCo_2_O_4_/C1 sample.

The XPS spectra in Fig. 5, S12, and S13 analyze the compositions and chemical status of the elements in the as-prepared materials. High-resolution spectra displayed signals from Ni 2p_3/2_ (Fig. 5a, S12a, S13a), Co 2p_3/2_ (Fig. 5b, S12b, S13b), O 1s (Fig. 5c, S12c, S13c), and C1s (Fig. 5d, S12d, S13d). By using z Shirley fitting method, the Ni 2p_3/2_ spectrum was well fitted considering two spin orbit double characteristic of Ni^2+^ and Ni^3+^. As shown in Fig. 5a, the Ni 2p_3/2_ shows two spin-orbit doubles characteristic of Ni^2+^ (854 eV) and Ni^3+^ (855.5 eV)^45–50^. In addition to the aforementioned two lines, the other four lines are associated with the satelites at the higher binding energy. The spectra of Co 2p_3/2_ cobalt were fitted with five lines, the peaks located at a binding energy of 779.6 eV and 782.3 eV, indicating the presence of Co^2+ 50,51^. The peak at 780.9 eV was attributed to Co^3+^ ions^50,51^. The other four lines are related to the satelites at the higher binding energy. The O 1s spectrum (Fig. 5c) can be divided into the main peaks which are identified the presence of metal oxides and Me-O bonds in the binding energy range of 529.6 eV. A binding energy value of 531.3 eV indicated the presence of O = C bonds and metal hydroxides, while 532.7 eV revealed O-C and/or adsorbed water bonds^52,53^. According to the high-resolution C1s spectrum (Fig. 5d) the carbon functionalities exist predominantly in the forms of C = C (sp^2^ hybrid carbon atom), C-C (sp^3^ hybrid carbon atom), C-O-C and/or C-OH, C = O and/or O-C-O, and O-C = O bond types at binding energies of 284.5, 285.0, 286.1, 287.3, and 289.0 eV, respectively^54,55^. The highest intensity of peak at 284.5 eV which is characteristic for sp^2^ bonded carbon atoms which presence of graphene in the structure is observed for NiCo_2_O_4_/C1 (Fig. 5d) where during the synthesis of hybrid commercial graphene was used. In the case of NiCo_2_O_4_/C3/S (Figure S13d) intensity of peak at 284.5 eV is lower because beside graphene in the structure is also carbon obtained from glucose. The lowest intensity peak at 284.5 eV was determined for NiCo_2_O_4_ (Figure S12d) where carbon impurities comes from residue from thermal urea treatment during the synthesis. At a binding energy of 291.5 eV, a spectrum arising from shake-up excitation was observed, indicating additional signals from sp^2^ carbon and its aromatic forms^54,56,57^.

The nitrogen adsorption/desorption isotherms and pore size distributions of NiCo_2_O_4_ and NiCo_2_O_4_/C are displayed in Figure S14a and S14b. All samples present type IV isotherm patterns, which are characteristic of both micropores and mesopores^58^. Their corresponding BET surface areas are shown in Table 1. It can be found that the BET of NiCo_2_O_4_ is only 25 m^2^ g^−1^, which can be increased to 105 m^2^ g^−1^ when the hybrid sample NiCo_2_O_4_/C3/N, S contain carbon doped with two heteroatoms (sulfur and nitrogen) for these samples the corresponding total pore volume increased from 0.157 to 0.202 cm^3^ g^−1^ correspondingly. Figure S14b shows that there is an obvious two peaks below 2 nm (at 0.5 nm and 1.5 nm) and two main peaks larger than 2 nm (at 3.8 nm and 5.9 nm) for all the hybrid samples, which indicates the forming of the micropores and mesopores in the structure after carbon modification of NiCo_2_O_4_.

**Table 1:** Structural parameters of micro-mesoporous NiCo_2_O_4_ and NiCo_2_O_4_/C hybrid series.

Sample	S_BET_^a^ (m^2^ g^−1^)	V_total_^b^ (cm^3^ g^−1^)	V_micro_^c^ (cm^3^ g^−1^)	V_meso_^d^ (cm^3^ g^−1^)
NiCo_2_O_4_	25	0.157	0.010	0.160
NiCo_2_O_4_/C1	61	0.157	0.026	0.147
NiCo_2_O_4_/C2	26	0.137	0.011	0.134
NiCo_2_O_4_/C3	77	0.192	0.032	0.109
NiCo_2_O_4_/C3/N, S	105	0.202	0.045	0.188
NiCo_2_O_4_/C3/S	93	0.209	0.040	0.193

^a^ S_BET_ – specific surface area; ^b^ V_total_ – single point pore volume (measured at the maximum partial pressure); ^c^ V_micro_ – volume of micropores; ^d^ V_meso_ – volume of mesopores.

The total pore volume between of 0.192 and 0.209 is for samples with C3 carbon and these values are much larger than the hybrids with C1 and C2. Additional mesopores (> 2 nm) can also be observed in the insert of Figure S14b for NiCo_2_O_4_/C3/N, S, while the mesopores in series with C3 aren’t presented on NiCo_2_O_4_/C3 and NiCo_2_O_4_/C3/S. The surface of the electrode materials significantly influences the catalytic properties for the hydrogen and oxygen evolution reactions, providing space for active sites conducive to mass transfer/electrochemical reactions.

## Electrochemical efficiency

To activate the obtained catalytic materials, 100 cycles of cyclic voltammetry (CV) were performed. The results of the 1st and 100th cycles are included in the Supplementary Information (Figure S15). As can be observed, the materials differ before and after the cycles. After 100 cycles, the Faradaic reactions associated with the redox reactions of the metal centers are not as prominent. This reduction in visibility of the redox reactions can be attributed to the possible stabilization and passivation of the active sites over the course of repeated cycling. The repeated cycling may lead to the formation of stable oxide layers or other structural changes in the materials, which can diminish the electrochemical activity of the metal centers by preventing their direct participation in redox processes.

**Fig. 6:**
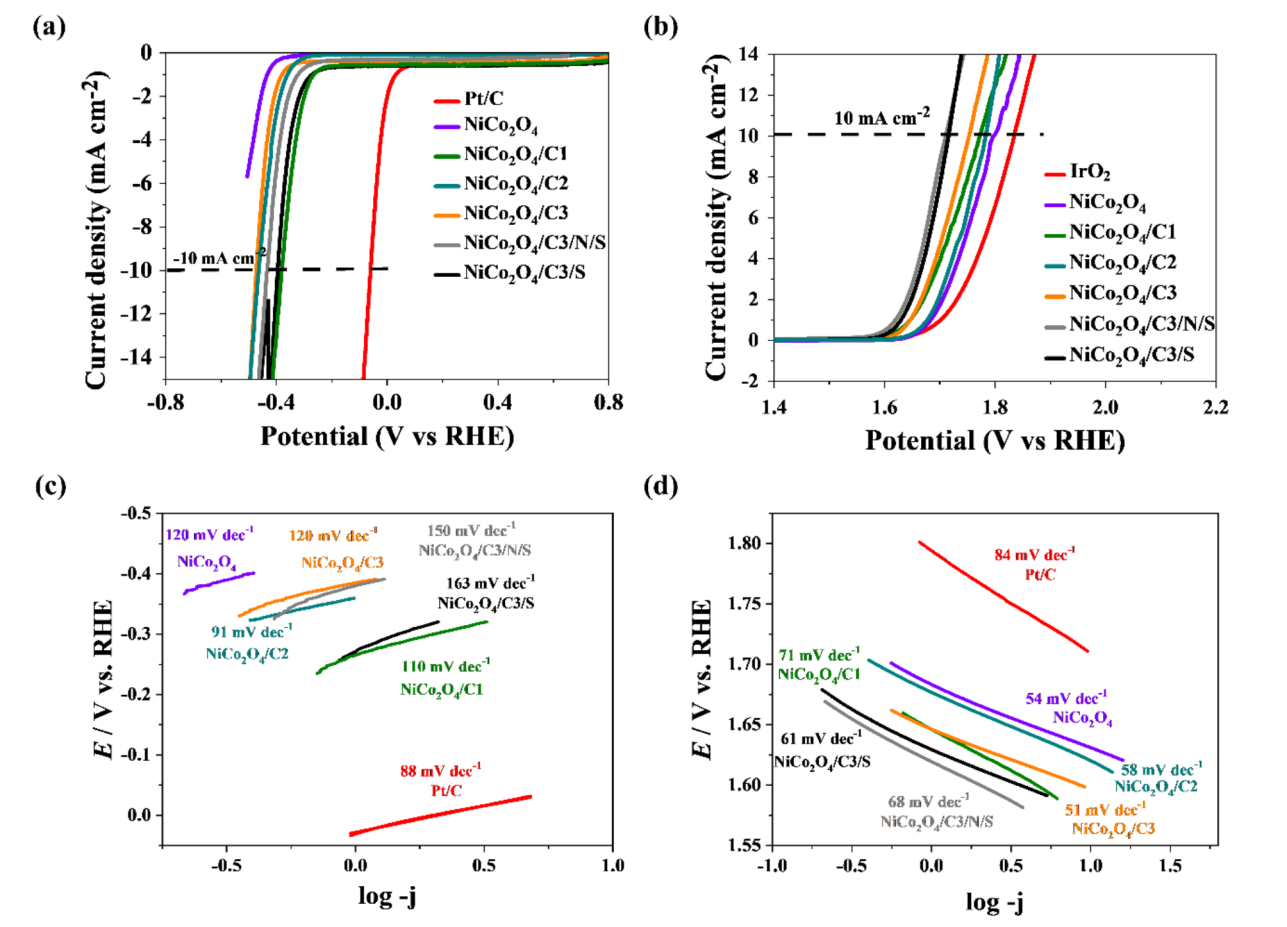
Linear voltammetry (LSV) curves for (**a**) hydrogen evolution reaction, (**b**) oxygen evolution reaction measured in 1 M KOH electrolyte solution. (**c**) Tafel slope plots for the HER profiles, (**d**) Tafel slope plots for the OER profiles.

Hydrogen evolution reaction (HER) assessments were carried out on synthesized NiCo_2_O_4_, as well as NiCo_2_O_4_/carbon material catalysts, within an alkaline electrolyte. Moreover, additional investigations were conducted on hybrid catalysts where carbon was doped with sulfur, as well as hybrid catalysts containing both sulfur and nitrogen. These supplementary analyses aimed to explore the impact of heteroatom incorporation on the catalytic performance. Figure 6a illustrates the linear sweep voltammetry (LSV) curves of the synthesized materials, juxtaposed with commercial Pt/C for reference. Notably, the hybrid catalysts exhibited significantly enhanced HER activity in comparison to the carbon-free catalyst. The augmentation of metal oxides with carbon has been widely recognized for its capacity to boost the efficiency of the hydrogen evolution reaction (HER). Numerous studies detailed in the literature have elucidated the multifaceted factors contributing to this enhancement. Notably, carbon, especially in the form of carbon nanomaterials, exhibits exceptional electrical conductivity. This incorporation of carbon into metal oxides serves to ameliorate the overall electrical conductivity of the composite material. The improved conductivity facilitates swifter electron transfer during the HER process, as extensively documented in previous research studies^59,60^. Furthermore, carbon-based materials, owing to their high surface area and porous structure, introduce additional catalytic sites. This characteristic expands the available active surface area for HER, thereby fostering more efficient electrochemical reactions^61,62^. The synergistic effects arising from the amalgamation of metal oxides and carbon play a pivotal role in elevating the overall catalytic performance. In this collaborative dynamic, carbon operates as a promoter, expediting charge transfer and aiding in the adsorption of reaction intermediates^63,64^. Simultaneously, the metal oxides contribute by furnishing the requisite catalytic active sites. This synergy underscores the intricate interplay between the two components, emphasizing their cooperative role in enhancing the catalytic efficacy of the composite material. Moreover, the positive influence of heteroatoms in carbon on the efficiency of the hydrogen evolution reaction has been noted. The introduction of heteroatoms, such as sulfur or sulfur/nitrogen, into carbon structures has been observed to enhance the catalytic performance in HER. The presence of heteroatoms introduces unique electronic configurations and active sites in the carbon matrix, contributing to improved catalytic activity. Heteroatoms in carbon play a crucial role by altering the electronic structure and creating defects in the carbon lattice. This modification leads to increased reactivity and affinity for intermediate species during the HER. Additionally, the introduction of heteroatoms can modify the surface chemistry of the carbon material, influencing the adsorption and desorption kinetics of reactants, further enhancing the overall catalytic efficiency^65–67^. According to linear sweep voltammograms (LSVs) in Fig. 6a the overpotential values at j_HER_ = 10 mA cm^− 2^ and onset potential for all electrodes are summarized in Table 2. From the data, it is evident that the Pt/C electrode exhibits the lowest overpotential of 61 mV, which is expected due to the high catalytic activity of platinum. Among the NiCo_2_O_4_ -based electrodes, the following observations can be made: (i) the pristine NiCo_2_O_4_ electrode does not achieve the required current density, indicating insufficient catalytic activity for HER, (ii) the NiCo_2_O_4_/C1, NiCo_2_O_4_C2, and NiCo_2_O_4_/C3 electrodes exhibit overpotentials of 378 mV, 468 mV, and 472 mV respectively. This indicates that the addition of carbon (C) improves the catalytic performance, with the NiCo_2_O_4_/C1 electrode showing the best improvement, (iii) The doping of the NiCo_2_O_4_/C3 electrode with nitrogen and sulfur (N/S) and sulfur alone (S) further enhances the HER performance, reducing the overpotential to 435 mV and 395 mV, respectively. These results highlight that while the overpotential values of the NiCo_2_O_4_-based electrodes are higher than that of the Pt/C electrode, the modifications with carbon and heteroatom doping significantly enhance the HER performance. Particularly, the NiCo_2_O_4_/C3/S electrode demonstrates the most notable improvement, suggesting that sulfur doping is particularly effective in enhancing the catalytic activity for HER. For comparison, we have compiled the overpotential results from the literature for platinum-based catalysts in Table S2. This table provides a clear benchmark for evaluating the performance of our catalysts against established platinum-based materials. In summary, while platinum-based catalysts remain superior in terms of overpotential, our study shows that with appropriate modifications, NiCo_2_O_4_-based electrodes can achieve substantial improvements in catalytic performance, making them viable candidates for cost-effective and efficient HER catalysts.

Based on the data presented in Fig. 6c, the Tafel slopes calculated for the hybrid catalysts fall within the range of 91–163 mV dec^**−**1^, which is higher than that of the commercial Pt/C catalyst (88 mV dec^**−**1^), but lower than the catalytic material without carbon. However, for materials containing heteroatom additives, an increase in the Tafel slope has been observed compared to the compound containing NiCo_2_O_4_ and carbon without heteroatoms in the hydrogen evolution reaction. The introduction of heteroatoms alters the electronic structure of the carbon material, leading to changes in the reaction kinetics. The presence of heteroatoms can introduce additional active sites, modify the adsorption energies of reaction intermediates, and impact the overall reaction mechanism. These modifications, in turn, influence the Tafel slope, reflecting the rate at which the electrochemical reaction proceeds. It is noteworthy that despite the observed increase in the Tafel slope for materials with heteroatom additives, they exhibit a lower onset potential. This seeming behavior suggests a complex interplay between the heteroatoms and the carbon matrix, influencing both the reaction kinetics and the initiation of the hydrogen evolution reaction.

**Table 2:** The HER parameters and Tafel slopes for the obtained catalysts.

Electrode	Overpotential (mV)*	Onset potential (mV)	b (mV dec^−1^)
Pt/C	61	-36	88
NiCo_2_O_4_	Does not achieve	-404	120
NiCo_2_O_4_/C1	378	-258	110
NiCo_2_O_4_/C2	468	-339	91
NiCo_2_O_4_/C3	472	-364	112
NiCo_2_O_4_/C3/N/S	435	-296	150
NiCo_2_O_4_/C3/S	395	-265	163

*Overpotential to achieve a current density of -10 mA cm^−2^.

Oxygen evolution reaction (OER) measurements were also conducted to assess the bifunctionality of the synthesized catalysts. Notably, all tested materials exhibited superior hydrogen evolution activity compared to the benchmark commercial compound, IrO_2_. In this context, the positive impact of carbon presence was similarly observed (Fig. 6b). Furthermore, catalysts incorporating heteroatoms demonstrated the most favorable performance among all tested catalysts. However, akin to the observations in the hydrogen evolution reaction (HER), a slight increase in the Tafel slope was noted for catalysts containing heteroatoms (Fig. 6d). It is crucial to emphasize that, despite this modest change in the Tafel slope, the obtained OER results were highly favorable for all materials, showcasing impressive performance in terms of Tafel slope, overpotential, and onset potential. The including carbons played a pivotal role in enhancing the catalytic efficiency of the materials. These heteroatoms not only contributed to modifying the electronic structure and creating active sites but also influenced the reaction kinetics during the oxygen evolution reaction. The overall bifunctional behavior of the catalysts underscores the promising potential of these materials for applications in energy conversion and storage technologies. All the determined results for the oxygen evolution reaction have been compiled and summarized in Table 3. To assess the sustained performance of the fabricated electrodes over an extended period, chronopotentiometric tests were conducted at a constant current density of -10 mA cm^−2^ (HER) and + 10 mA cm^−2^ (OER), as depicted in Figure S16.

**Table 3:** The OER parameters and Tafel slopes for the obtained catalysts.

Electrode	Overpotential (mV) *	Onset potential (mV)	b (mV dec^−1^)
IrO_2_	1830	420	84
NiCo_2_O_4_	1796	410	54
NiCo_2_O_4_/C1	1771	400	71
NiCo_2_O_4_/C2	1784	370	58
NiCo_2_O_4_/C3	1752	370	51
NiCo_2_O_4_/C3/N/S	1711	350	68
NiCo_2_O_4_/C3/S	1713	360	61

* Overpotential to achieve a current density of -10 mA cm^−2^.

The results indicate commendable stability of overpotential for nearly all electrodes under the applied polarization current. In the case of HER, the pure Pt/C electrode exhibited a discernible shift in overpotential, escalating from 0 to 300 mV within the 25 h testing duration. Conversely, the remaining electrodes demonstrated overpotential slight fluctuations during the same timeframe. For OER, all catalysts demonstrated similar stability; however, differences were observed in the oxygen evolution overpotential, with the commercial material IrO₂ showing the highest overpotential. These findings underscore the enduring stability of most electrodes, highlighting their potential suitability for sustained applications in HER and OER.

**Fig. 7:**
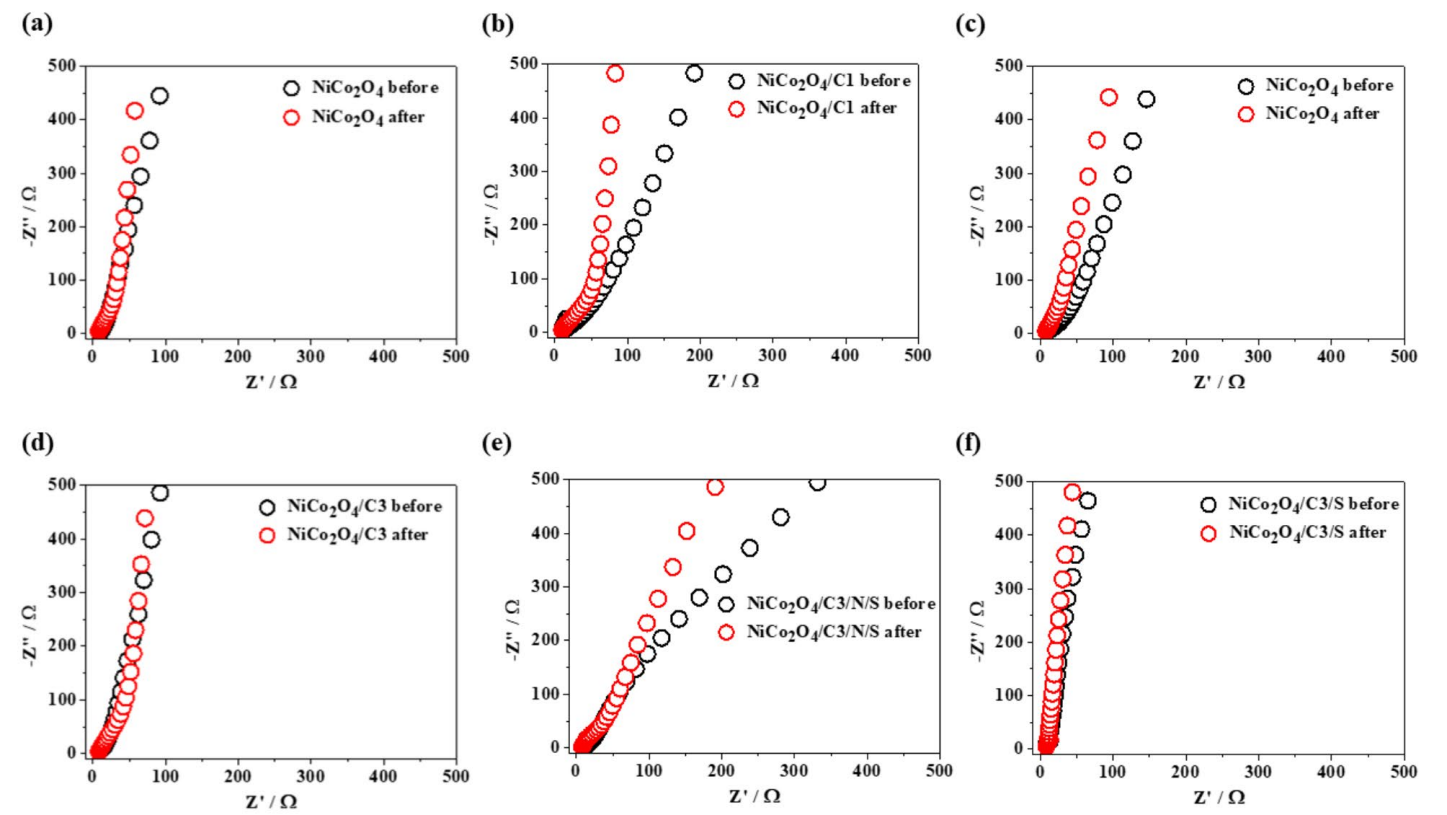
EIS spectra of (**a**) NiCo_2_O_4_, (**b**) NiCo_2_O_4_/C1, (**c**) NiCo_2_O_4_/C2, (**d**) NiCo_2_O_4_/C3, (**e**) NiCo_2_O_4_/C3/N/S, (**f**) NiCo_2_O_4_/C3/S before and after cycling stability test (HER and OER).

Figure 7 shows the corresponding Nyquist plots obtained within the 1–20 kHz frequency range at an open circuit potential. The electrochemical impedance spectra exhibit a semicircular feature in the high-frequency region and a straight line in the low-frequency region. In the high-frequency domain, the intersection of the curve with the real axis represents the series resistance (Rs), which includes the intrinsic resistance of the material, the ionic resistance of the electrolyte, and the interfacial resistance between the GC electrode and the deposited material. The diameter of the semicircle corresponds to the charge-transfer resistance (Rct). Both Rs and Rct values showed a slight increase after the electrochemical stability tests for HER and OER. The minor differences in the electrochemical impedance spectra indicate the stability of the materials after the electrochemical tests.

## Conclusions

The result of the present work was to obtain effective electrocatalysts for hydrogen evolution reactions and oxygen evolution reactions. Hybrid materials containing nickel cobaltite NiCo_2_O_4_ were proved to be bi-functional catalysts. Analysis of X-ray diffractograms confirmed the obtaining of hybrid materials containing high-purity NiCo_2_O_4_ spinel in their composition. Raman spectra confirmed that the materials doped with carbon material contain graphene in their structure. SEM-EDX analysis made it possible to confirm the chemical composition of the obtained catalysts, for which cobalt is the dominant component. The results of electrochemical studies confirmed that the addition of graphene in the catalyst has a beneficial effect on improving the catalytic properties in the HER reaction. The highest catalytic activity measured in 1 M KOH electrolyte was shown by the NiCo_2_O_4_/C1 catalyst, where the addition of graphene favorably affected the course of the hydrogen evolution reaction. In the OER reaction, the NiCo_2_O_4_ spinel without graphene addition showed better catalytic activity. The addition of nitrogen and sulfur heteroatoms adversely affected the properties of the electrocatalysts in the OER reaction, these materials showed lower overpotential values than for NiCo_2_O_4_ and hybrid NiCo_2_O_4_/C samples. Nitrogen and sulfur, being heteroatoms, can introduce new electronic states within the catalyst material. These changes in the electronic structure may influence the redox properties and electron transfer kinetics during the OER, contributing to decreased catalytic efficiency. These findings collectively underscore the significance of graphene addition in enhancing catalytic performance in the HER, while emphasizing the nuanced influence of heteroatoms in the OER. The best material for the HER reaction was characterized by Tafel slopes of 91 mV dec⁻¹, overpotential of 468 mV, and onset potential of -339 mV. For the OER reaction, the material showed Tafel slopes of 51 mV dec⁻¹, overpotential of 1752 mV, and onset potential of 370 mV.

## Materials and methods

### Synthesis of NiCo2O4 and their hybrid with carbon

NiCo_2_O_4_ was obtained using Ni(NO_3_)_2_*6H_2_O (0.58 g) and Co(NO_3_)_2_*6H_2_O (1.75 g) in a mass ratio of 1:3, to which 50 mL of distilled water was added, the whole mixture was stirred for 5 min. using a magnetic stirrer. Next, 1.92 g of urea was added and stirring continued for about 10 min. The next step was to conduct the hydrothermal process at 150 °C for 10 h. The product, after being removed from the reactor, was centrifuged, washed on a Büchner funnel with a nylon filter with distilled water and ethanol and dried in a laboratory electric oven for about 12 h at 60 °C in ambient air. The resulting mass was subjected to an annealing process, which was carried out in an air atmosphere at 450 °C with a heating rate of 3 °C/min, after reaching the desired temperature it was annealed for a period of 8 h.

To obtain hybrid materials, carbon material was added during the synthesis of NiCo_2_O_4_ catalyst. The catalyst described above was enriched using three selected carbon materials. For the first hybrid material denoted NiCo_2_O_4_/C1, 0.05 g of commercial graphene nanoplatelets (purchased from Sigma Aldrich) with an initial specific surface area of 750 m^2^ g^−1^ denoted C1 were used during the synthesis. For the next two samples denoted NiCo_2_O_4_/C2 and NiCo_2_O_4_/C3, carbon materials C2 (0.05 g) and C3 (0.05 g) were used and synthesized as described below. Sample C2 was prepared by grinding 2 g of glucose with 2 g of FeCl_3_*6H_2_O in a mortar. Then 5 mL of distilled water was added and the grinding continued, after which it was transferred to a porcelain boat and another 5 mL of distilled water was added. The resulting mass was dried for 24 h at 80 °C. The carbonization process was carried out under a nitrogen atmosphere at 700 °C, the heating rate was 5 °C min^−1^, and the annealing time was equal to 6 h. After this process, the product was washed with concentrated hydrochloric acid. The C3 material was prepared analogously, with the difference that during the synthesis 2 g of graphene GF750 was additionally added. The course of synthesis was analogous to that of sample C2. In order to introduce, in addition to carbon C3 (0.05 g), additional sulfur and nitrogen heteroatoms, thiourea (1.63 g), or methylsulfonylmethane (1.47 g) were added during the synthesis of the samples, and the samples were labeled NiCo_2_O_4_/C3/N/S and NiCo_2_O_4_/C3/S, respectively. All hybrid materials were annealed for four hours in an inert N_2_ gas atmosphere.

### Solid state physics techniques

A 1430 VP scanning electron microscope, produced by LEO Electron Microscopy Ltd, was used to determine the surface morphology of the sample, while an X-ray spectrometer (EDX) Quantax 200 with an XFlash 4010 detector, produced by Bruker AXS, was used to determine the elemental composition. Transmission electron microscopy (TEM) analyses were conducted using a Tecnai TF20 X-TWIN (FEG) microscope (FEI) operating at an accelerating voltage of 200 kV. The TEM samples were prepared by drop casting the particles suspended in isopropyl alcohol onto carbon-coated copper TEM grids. The electron diffraction patterns were examined using ProcessDiffraction software. A Philips X “Pert instrument with an X “Celerator Scientific detector was used to determine crystallographic parameters. A Senterra dispersive spectrometer from Bruker Optik was used to measure Raman scattering experiment. N_2_ adsorption-desorption analysis at -196 °C was carried out by automatic physical adsorption ASAP 2020 Plus analyzer. The specific surface area was determined by the Brunauer−Emmett−Teller (BET) method, while microporous volume and mesoporous volume was determined by t-plot and density functional theory (DFT) methods, respectively. XPS analysis was carried out with a PHI VersaProbeII instrument (ULVAC-PHI, Chigasaki, Japan) using a focused monochromatic X-ray beam of the Al Kα line (1486.6 eV). The beam was focused to a spot of 100 μm diameter scanning a 400 μm x 400 μm area on the sample surface. For each measurement spot, one broad-band spectrum (0–1200 eV) with low resolution (0.5 eV) and high-resolution spectra (0.1 eV) were measured in the areas of line occurrence: C 1s, O 1s, Co 2p and Ni 2p. During the measurements, surface charge neutralization was applied by irradiating the surface with a beam of low-energy electrons (1 eV) and ions (7 eV Ar^+^) to ensure a constant surface potential despite the emitted photoelectrons. The vacuum value during the measurement oscillated around 3 × 10^−9^ mbar. Matching of spectral lines was carried out using the PHI Multipak program (v.9.9.3) subtracting the background using the Shirley method^68^.

## Electrochemical measurements

The catalytic activity of the obtained catalysts was studied for the HER reaction and the OER reaction using the linear voltammetry (LSV) method. The measurements were carried out using an Autolab electrochemical analyzer (PGSTAT128N, the Netherlands). The measurement system consisted of three electrodes, and tests were conducted in a 1 M KOH electrolyte solution. The reference electrode was Ag/AgCl in 3 M KCl, a platinum plate was used as the counter-electrode. The working electrode was the obtained catalysts applied to an electrode made of glassy carbon with a diameter of 3 mm. To prepare the ink, 3 mg of catalyst was weighed, then 0.75 mL of distilled water, 0.2 mL of isopropyl alcohol, and 0.05 mL of Nafion (5% solution) were added. The prepared mixture was sonified in an ultrasonic bath for a period of 30 min. Then 5.3 µl of the catalyst solution was applied to a 3 mm glassy carbon working electrode, followed by drying at 60 °C for a period of several minutes to evaporate the solvent. LSV measurements were performed at a scan rate of 1 mV s^−1^, then at a current density of -10 mA cm^−2^ for the hydrogen evolution reaction and 10 mA cm^−2^ for the oxygen evolution reaction, the values of the achieved potential for the corresponding catalyst were read. The stability of the obtained materials was measured over a period of 25 h. Electrochemical Impedance Spectroscopy (EIS) measurements before and after cycling stability test were performed over a frequency range of 20 kHz to 1 Hz, using a voltage amplitude of 10 mV at an open circuit potential.

## Electronic supplementary material

Below is the link to the electronic supplementary material.

Supplementary Material 1

## Data Availability

The datasets generated and/or analysed during the current study are available in the BRIDGE OF KNOWLEDGE repository (https://mostwiedzy.pl/pl/open-research-data/x-ray-diffraction-spectra-of-the-nico2o4-modified-by-carbon,220103513213198-0).

## References

[1] Khan, R. et al. Role of perovskites as a bi-functional catalyst for electrochemical water splitting: a review. *Int. J. Energy Res. ***44**, 9714–9747. 10.1002/er.5635 (2020).

[2] Martindale, B. C. M. & Reisner, E. Bi-functional Iron-only electrodes for efficient water splitting with enhanced Stability through in situ Electrochemical Regeneration. *Adv. Energy Mater. ***6**, 1502095. 10.1002/aenm.201502095 (2016).

[3] Zhang, R. et al. In situ engineering bi-metallic phospho-nitride bi-functional electrocatalysts for overall water splitting. *Applied Catalysis B: Environmental ***254**, 414–423 (2019).

[4] Sun, L., Luo, Q., Dai, Z. & Ma, F. J. C. C. R. Material libraries for electrocatalytic overall water splitting. *Coordination Chemistry Reviews ***444**, 214049 (2021).

[5] Koponen, J. Review of water electrolysis technologies and design of renewable hydrogen production systems. (2015).

[6] Ayers, K. E. et al. Research advances towards low cost, high efficiency PEM electrolysis. *ECS Trans. ***33**, 3 (2010).

[7] You, B. & Sun, Y. J. Innovative strategies for electrocatalytic water splitting. *O c r*. **51**, 1571–1580 (2018). 10.1021/acs.accounts.8b00002 29537825

[8] Xu, W. & Wang, H. Earth-abundant amorphous catalysts for electrolysis of water. *Chin. J. Catal. ***38**, 991–1005. 10.1016/S1872-2067(17)62810-9 (2017).

[9] Wang, Y. Z., Yang, M., Ding, Y. M., Li, N. W. & Yu, L. J. Recent advances in complex hollow electrocatalysts for water splitting. *F M*. **32**, 2108681 (2022).

[10] Jiang, J., Zhou, X. L., Lv, H. G., Yu, H. Q. & Yu, Y. Bimetallic-based electrocatalysts for oxygen evolution reaction. *Adv. Funct. Mater. ***33**, 2212160 (2023).

[11] Alom, M. S., Kananke-Gamage, C. C. & Ramezanipour, F. Perovskite oxides as electrocatalysts for hydrogen evolution reaction. *ACS Omega*. **7**, 7444–7451 (2022). 35284721 10.1021/acsomega.1c07203 PMC8908488

[12] Zhuang, L. et al. Sulfur-modified oxygen vacancies in iron–cobalt oxide nanosheets: enabling extremely high activity of the oxygen evolution reaction to achieve the industrial water splitting benchmark. *Angew. Chem. Int. Ed. ***59**, 14664–14670 (2020). 10.1002/anie.202006546 32495475

[13] Shi, W. et al. Ce-substituted spinel CuCo_2_O_4_ quantum dots with high oxygen vacancies and greatly improved electrocatalytic activity for oxygen evolution reaction. *Inorg. Chem. ***60**, 19136–19144 (2021). 34839658 10.1021/acs.inorgchem.1c02931

[14] Zuo, P. et al. P co-doped Ni/Mo-based multicomponent electrocatalysts in situ decorated on ni foam for overall water splitting. *J. Colloid Interface Sci. ***645**, 895–905 (2023). 37178566 10.1016/j.jcis.2023.04.166

[15] Xia, L. et al. Defect and interface engineering of templated synthesis of hollow porous Co_3_O_4_/CoMoO_4_ with highly enhanced electrocatalytic activity for oxygen evolution reaction. *Chem. Eng. J. ***452**, 139250 (2023).

[16] Zhang, W. et al. Porous Pd/NiFeOx nanosheets enhance the pH-universal overall water splitting. *Adv. Funct. Mater. ***31**, 2107181 (2021).

[17] Olowoyo, J. O. & Kriek, R. J. Recent progress on bimetallic-based spinels as electrocatalysts for the oxygen evolution reaction. *Small*. **18**, 2203125 (2022). 10.1002/smll.202203125 35996806

[18] Rijith, S., Abhilash, S., Sarika, S., Sumi, V. & Sreekala, C. J. I. Tuning of electrocatalytic activity of mixed metal dichalcogenides supported NiMoP coatings for alkaline hydrogen evolution reaction. *International Journal of Hydrogen Energy ***48**, 5783–5800 (2023).

[19] Tian, L. et al. Carbon quantum dots modulated NiMoP hollow nanopetals as efficient electrocatalysts for hydrogen evolution. *Industrial & Engineering Chemistry Research*. **58**, 14098–14105 (2019).

[20] Wang, S. et al. Rational assembly of the NiMoP/NiCoZn heterostructure electrocatalyst for the hydrogen evolution reaction at high current densities. *The Journal of Physical Chemistry C ***127**, 958–967 (2023).

[21] Gao, X. et al. Hierarchical NiCo_2_O_4_ hollow microcuboids as bifunctional electrocatalysts for overall water-splitting. *Angew. Chem. Int. Ed. ***55**, 6290–6294 (2016). 10.1002/anie.201600525 27061909

[22] Béjar, J., Álvarez-Contreras, L., Ledesma-García, J., Arjona, N. & Arriaga, L. Electrocatalytic evaluation of Co_3_O_4_ and NiCo_2_O_4_ rosettes-like hierarchical spinel as bifunctional materials for oxygen evolution (OER) and reduction (ORR) reactions in alkaline media. *J. Electroanal. Chem. ***847**, 113190 (2019).

[23] Dubal, D. P., Gomez-Romero, P., Sankapal, B. R. & Holze, R. Nickel cobaltite as an emerging material for supercapacitors: an overview. *Nano Energy*. **11**, 377–399 (2015).

[24] Umeshbabu, E., Rajeshkhanna, G., Justin, P. & Rao, G. R. NiCo_2_O_4_/rGO hybrid nanostructures for efficient electrocatalytic oxygen evolution. *J. Solid State Electrochem. ***20**, 2725–2736 (2016).

[25] Zhao, Q., Yan, Z., Chen, C. & Chen, J. Spinels: controlled preparation, oxygen reduction/evolution reaction application, and beyond. *Chem. Rev. ***117**, 10121–10211 (2017). 28745484 10.1021/acs.chemrev.7b00051

[26] Debata, S., Patra, S., Banerjee, S., Madhuri, R. & Sharma, P. K. Controlled hydrothermal synthesis of graphene supported NiCo_2_O_4_ coral-like nanostructures: An efficient electrocatalyst for overall water splitting. *Appl. Surf. Sci. ***449**, 203–212 (2018).

[27] Umeshbabu, E., Hari Krishna Charan, P. & Justin, P. Ranga Rao, G. Hierarchically organized NiCo_2_O_4_ microflowers anchored on multiwalled carbon nanotubes: Efficient bifunctional electrocatalysts for oxygen and hydrogen evolution reactions. *ChemPlusChem*. **85**, 183–194 (2020).

[28] Liu, Z. Q., Cheng, H., Li, N., Ma, T. Y. & Su, Y. Z. ZnCo_2_O_4_ quantum dots anchored on nitrogen-doped carbon nanotubes as reversible oxygen reduction/evolution electrocatalysts. *Adv. Mater. (Deerfield Beach Fla)*. **28**, 3777–3784 (2016). 10.1002/adma.201506197 26996677

[29] Tu, J., Zhang, M., Li, M., Li, J. & Zhi, L. Phosphorus-doped nickel cobalt oxide (NiCo_2_O_4_) wrapped in 3D hierarchical hollow N-doped carbon nanoflowers as highly efficient bifunctional electrocatalysts for overall water splitting. *J. Colloid Interface Sci.* (2024). 10.1016/j.jcis.2024.04.156 38678880

[30] Donya, H., Aman, S., Ahmad, N., Farid, H. M. T. & Taha, T. A. M. Development of SnCo_2_O_4_ spinel supported on the rGO nanosheet with the improved electrochemical performance of OER activity. *Int. J. Hydrog. Energy*. **51**, 436–447 (2024).

[31] Liu, M., Zhang, R. & Chen, W. Graphene-supported nanoelectrocatalysts for fuel cells: Synthesis, properties, and applications. *Chem. Rev. ***114**, 5117–5160 (2014). 24666160 10.1021/cr400523y

[32] Rebekah, A., Kumar, E. A., Viswanathan, C. & Ponpandian, N. Effect of cation substitution in MnCo_2_O_4_ spinel anchored over rGO for enhancing the electrocatalytic activity towards oxygen evolution reaction (OER). *Int. J. Hydrog. Energy*. **45**, 6391–6403 (2020).

[33] Wu, Z., Li, P., Qin, Q., Li, Z. & Liu, X. N-doped graphene combined with alloys (NiCo, CoFe) and their oxides as multifunctional electrocatalysts for oxygen and hydrogen electrode reactions. *Carbon*. **139**, 35–44 (2018).

[34] Wu, X., Li, S., Wang, B., Liu, J. & Yu, M. Graphene foam supported multilevel network-like NiCo_2_S_4_ nanoarchitectures for robust lithium storage and efficient ORR catalysis. *New J. Chem. ***41**, 115–125 (2017).

[35] Gebreslase, G. A., Martínez-Huerta, M. V., Sebastián, D. & Lázaro, M. J. Transformation of CoFe_2_O_4_ spinel structure into active and robust CoFe alloy/N-doped carbon electrocatalyst for oxygen evolution reaction. *J. Colloid Interface Sci. ***625**, 70–82 (2022). 35714410 10.1016/j.jcis.2022.06.005

[36] Li, Z., Li, B., Chen, J., Pang, Q. & Shen, P. J. I. J. o. H. E. Spinel NiCo_2_O_4_ 3-D nanoflowers supported on graphene nanosheets as efficient electrocatalyst for oxygen evolution reaction. *Int. J. Hydrogen Energy*. **44**, 16120–16131 (2019).

[37] Tong, X. et al. Mesoporous NiCo_2_O_4_ nanoplates on three-dimensional graphene foam as an efficient electrocatalyst for the oxygen reduction reaction. *Healthcare, and Environmental Applications* **8**, 28274–28282 (2016). 10.1021/acsami.5b10044 26796978

[38] Debata, S. et al. A. S. S. Controlled hydrothermal synthesis of graphene supported NiCo_2_O_4_ coral-like nanostructures: an efficient electrocatalyst for overall water splitting. *Applied Surface Science*. **449**, 203–212 (2018).

[39] Lee, D. U., Kim, B. J. & Chen, Z. J. A. one-pot synthesis of a mesoporous NiCo_2_O_4_ nanoplatelet and graphene hybrid and its oxygen reduction and evolution activities as an efficient bi-functional electrocatalyst. *J. Mater. Chem. A*. **1**, 4754–4762 (2013).

[40] Deokate, R. J. Chemically deposited NiCo_2_O_4_ thin films for electrochemical study. *ES Materials & Manufacturing*. **11**, 16–19 (2020).

[41] Nakate, U. T. & Kale, S. Microwave assisted synthesis and characterizations of NiCo_2_O_4_ nanoplates and electrical, magnetic properties. *Materials Today: Proceedings*. **3**, 1992–1998 (2016).

[42] Babu, G. A., Ravi, G., Hayakawa, Y., Karaikudi, T. N. K. & J. I. J. S. E. A. Surfactant assisted growth and optical studies of NiCo_2_O_4_ nanostructures through microwave heating method. *International Journal of Science and Engineering Applications*. **1**, 17–20 (2014).

[43] Mohamed, Z., Zhang, G., He, B. & Fan, Y. Heterostructure necklace-like NiO-NiCo_2_O_4_ hybrid with superior catalytic capability as electrocatalyst for Li-O_2_ batteries. *Engineered Science*. **17**, 231–241 (2021).

[44] Dudek, A., Szwej, D., Wita, H. & Pawlyta, M. Porównanie struktury kish grafitu, grafitu syntetycznego oraz grafitu naturalnego z wykorzystaniem nowoczesnych metod badawczych. *Zeszyt Studenckich Kół Naukowych*. **4**, 55 (2017).

[45] Liu, Z. et al. Significantly enhanced photocurrent density in NiCo_2_O_4_/aC/Si photoanode for water splitting. *Applied Surface Science*. **529**, 147155 (2020).

[46] Bhanuchandar, S. et al. Unravelling the role of cationic Ni^2+^ vacancies and Ni^3+^ ions in non-stoichiometric NiO: breakdown of anti-ferromagnetic ordering and large exchange bias. *Journal of Materials Science*. **58**, 13136–13153 (2023).

[47] Zhang, P. et al. Reinforcing Li-rich layer cathode via artificial surface reconstruction of spinel shell containing Ni^3+^. *Applied Surface Science*. **554**, 149626 (2021).

[48] Marco, J. et al. Characterization of the nickel cobaltite, NiCo_2_O_4_, prepared by several methods: an XRD, XANES, EXAFS, and XPS study. *Journal of Solid State Chemistry*. **153**, 74–81 (2000).

[49] Guo, Y. et al. XPS Analysis of FexNiyPS3 vdW Materials Used in the Hydrogen Evolution Processes. *ChemPhysChem*. e202400039 (2024). 10.1002/cphc.202400039 38526205

[50] Yewale, M. et al. Hydrothermally synthesized microrods and microballs of NiCo_2_O_4_ for supercapacitor application. *Ceramics International*. **48**, 21996–22005 (2022).

[51] Murtaza, A. et al. Role of divalent Co^2+^ and trivalent Tb^3+^ incorporation in ZnO nanocrystals: Structural, optical, photoluminescence properties and enhanced ferromagnetism. *Physica B: Condensed Matter*. **646**, 414287 (2022).

[52] DW, C. J. h. s. n. g. x. NIST Standard Reference Database 20, Version 3.4. *Reference fluid thermodynamic and transport properties*. (2003).

[53] Biesinger, M. C. et al. Resolving surface chemical states in XPS analysis of first row transition metals, oxides and hydroxides: Cr, Mn, Fe, Co and Ni. *Applied Surface Science*. **257**, 2717–2730 (2011).

[54] Beamson, G. J. I. *High Relution XPS of Organic Polymers. The Scienta ESCA 300 Database* (1992).

[55] Rouxhet, P. G., Genet, M. J. XPS analysis of bio-organic systems. **43**, 1453–1470 (2011).

[56] Briggs, D. *Surface Analysis of Polymers by XPS and Static SIMS* (Cambridge University Press, 1998).

[57] Genet, M. J., Dupont-Gillain, C. C. & Rouxhet, P. G. XPS analysis of biosystems and biomaterials. **177** (2008).

[58] Li, P. et al. Salt assisted fabrication of lignin-derived Fe, N, P, S codoped porous carbon as trifunctional catalyst for Zn-air batteries and water-splitting devices. **421**, 129704 (2021).

[59] Jiang, J. et al. Recent advances in metal oxide-based electrode architecture design for electrochemical energy storage. *Adv. Mater. ***24**, 5166–5180 (2012). 22912066 10.1002/adma.201202146

[60] Zhang, W. D., Xu, B. & Jiang, L. C. Functional hybrid materials based on carbon nanotubes and metal oxides. *J. Mater. Chem. ***20**, 6383–6391 (2010).

[61] Rodriguez-Reinoso, F. The role of carbon materials in heterogeneous catalysis. *Carbon*. **36**, 159–175 (1998).

[62] Levy, N. et al. The relationship of morphology and catalytic activity: a case study of iron corrole incorporated in high surface area carbon supports. *Carbon*. **158**, 238–243 (2020).

[63] Kwon, H. C. et al. Carbon monoxide as a promoter of atomically dispersed platinum catalyst in electrochemical hydrogen evolution reaction. *Journal of the American Chemical Society ***140**, 16198–16205 (2018). 30383962 10.1021/jacs.8b09211

[64] Murthy, A. P., Madhavan, J. & Murugan, K. Recent advances in hydrogen evolution reaction catalysts on carbon/carbon-based supports in acid media. *J. Power Sources*. **398**, 9–26 (2018).

[65] Qu, K. et al. Promotion of electrocatalytic hydrogen evolution reaction on nitrogen-doped carbon nanosheets with secondary heteroatoms. *ACS Nano*. **11**, 7293–7300 (2017). 28640994 10.1021/acsnano.7b03290

[66] Zhang, J. & Dai, L. Heteroatom-doped graphitic carbon catalysts for efficient electrocatalysis of oxygen reduction reaction. *ACS Catal. ***5**, 7244–7253 (2015).

[67] Jin, H. et al. Heteroatom-doped transition metal electrocatalysts for hydrogen evolution reaction. *ACS Energy Lett. ***4**, 805–810 (2019).

[68] Shirley, D. A. High-resolution X-ray photoemission spectrum of the valence bands of gold. **5**, 4709 (1972).

