# Supplementary material for: Combined effect of nitrogen-doped carbon and NiCo2O4 for electrochemical water splitting
Source: Sci Rep. 2024 Nov 6;14:26930. doi: 10.1038/s41598-024-74031-1 (PMC11541751; doi:10.1038/s41598-024-74031-1)
Supplement: Supplementary file 1 — Supplementary Material 1 [file 41598_2024_74031_MOESM1_ESM.docx]

**Supporting Information**

**Combined effect of nitrogen-doped carbon and NiCo_2_O_4_ for electrochemical water splitting**

Laura Kubińska ^1^, Mariusz Szkoda ^2,3^, Malgorzata Skorupska ^1^, Patrycja Grabowska ^1^, Marta Gajewska ^4^, Jerzy P. Lukaszewicz ^1,5^, Anna Ilnicka ^1,^*

^1^ Faculty of Chemistry, Nicolaus Copernicus University in Torun, Gagarina 7, 87-100 Torun, Poland

^2^ Faculty of Chemistry, Department of Chemistry and Technology of Functional Materials, Gdańsk University of Technology, Narutowicza 11/12, 80-233 Gdańsk, Poland

^3^ Advanced Materials Center, Gdańsk University of Technology, Narutowicza 11/12, 80-233 Gdańsk, Poland

^4^ Academic Centre for Materials and Nanotechnology, AGH University of Krakow, Mickiewicza 30, 30-059 Kraków, Poland

^5^ Centre for Modern Interdisciplinary Technologies, Nicolaus Copernicus University in Torun, Wilenska 4, 87-100 Torun, Poland.

***** Corresponding author. E-mail address: ailnicka@umk.pl


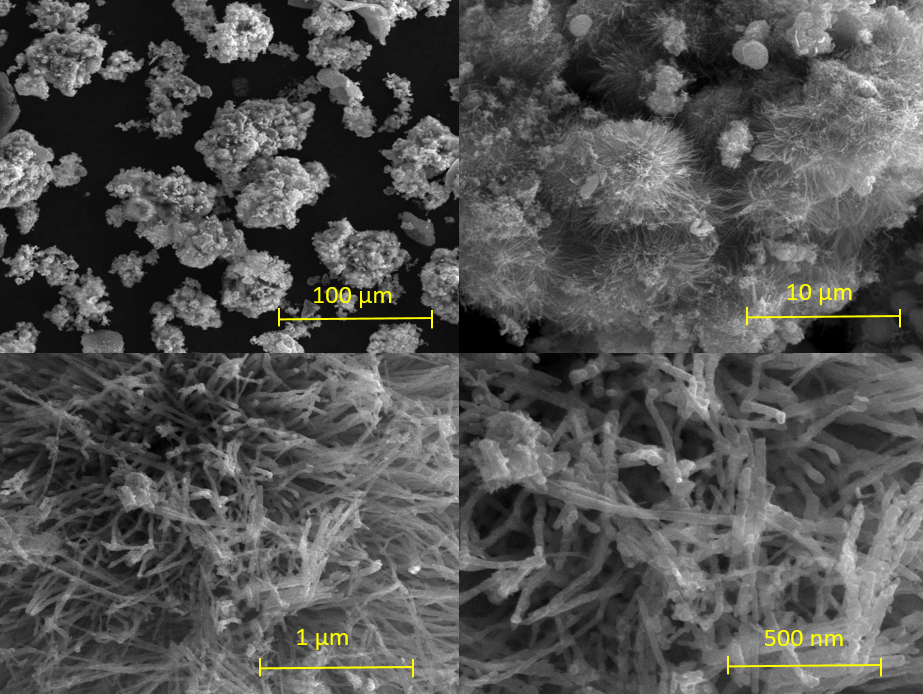


**Figure S1.** SEM images of samples NiCo_2_O_4_/C2 with different magnifications.

**
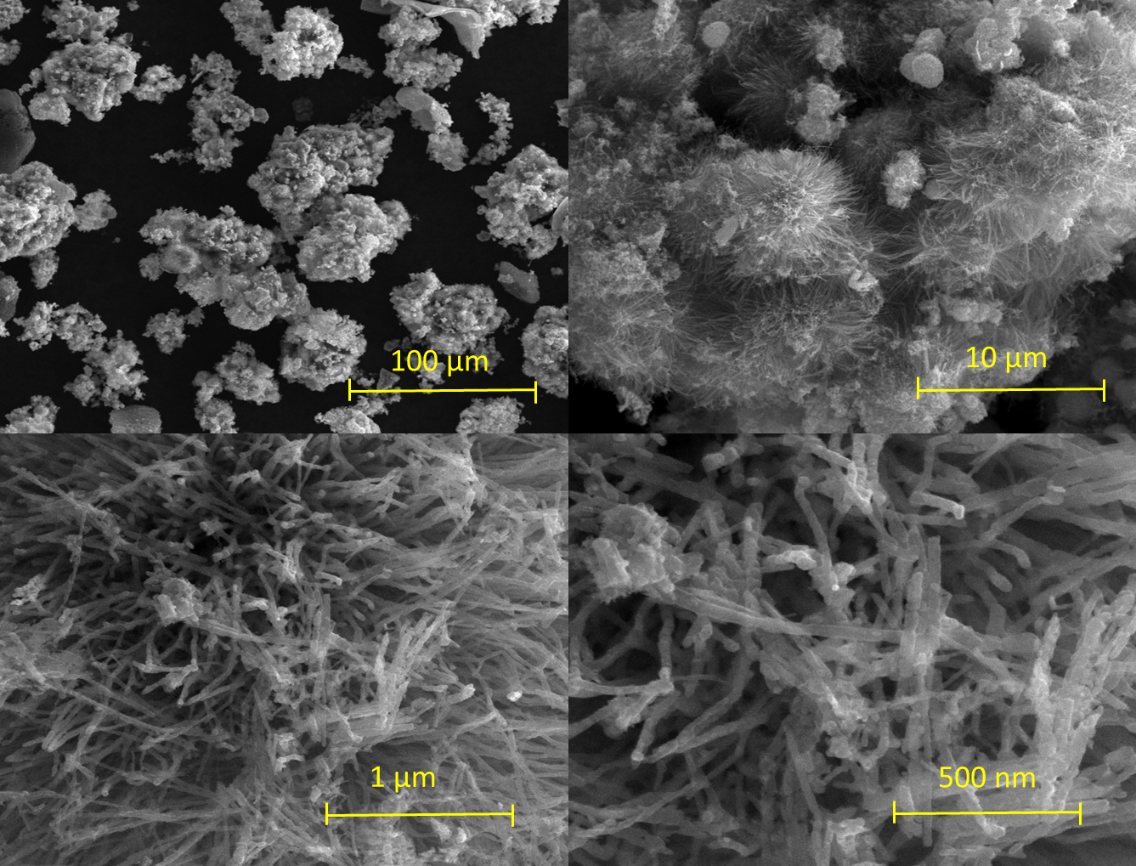
**

**Figure S2.** SEM images of samples NiCo_2_O_4_/C3 with different magnifications.


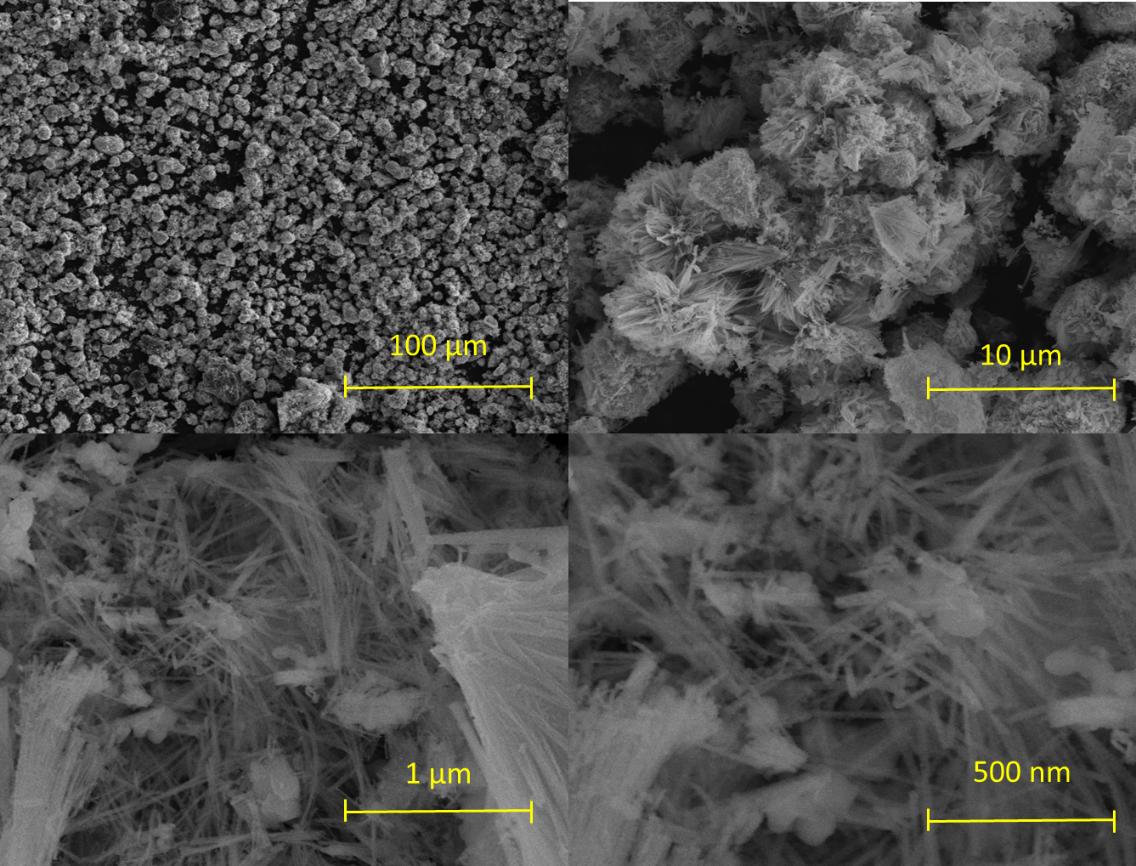


**Figure S3.** SEM images of samples NiCo_2_O_4_/C3/N/S with different magnifications.


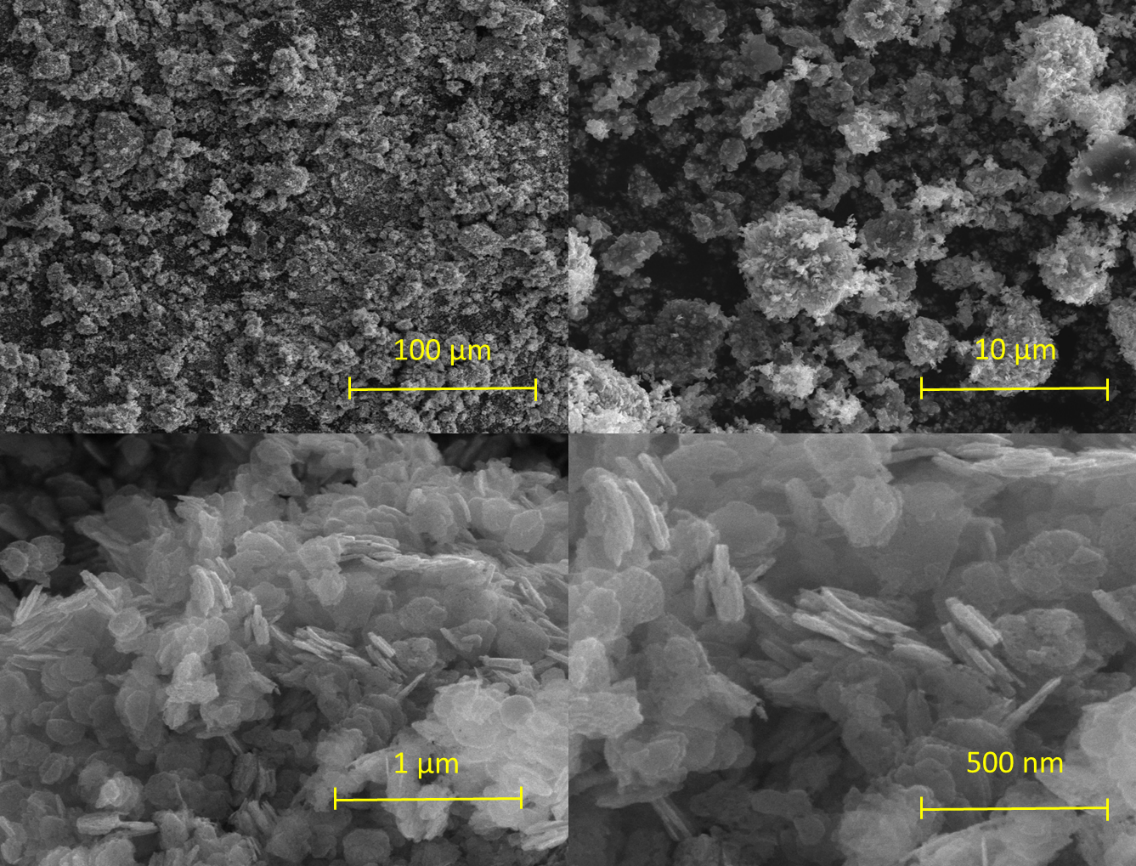


**Figure S4.** SEM images of samples NiCo_2_O_4_/C3/S with different magnifications.


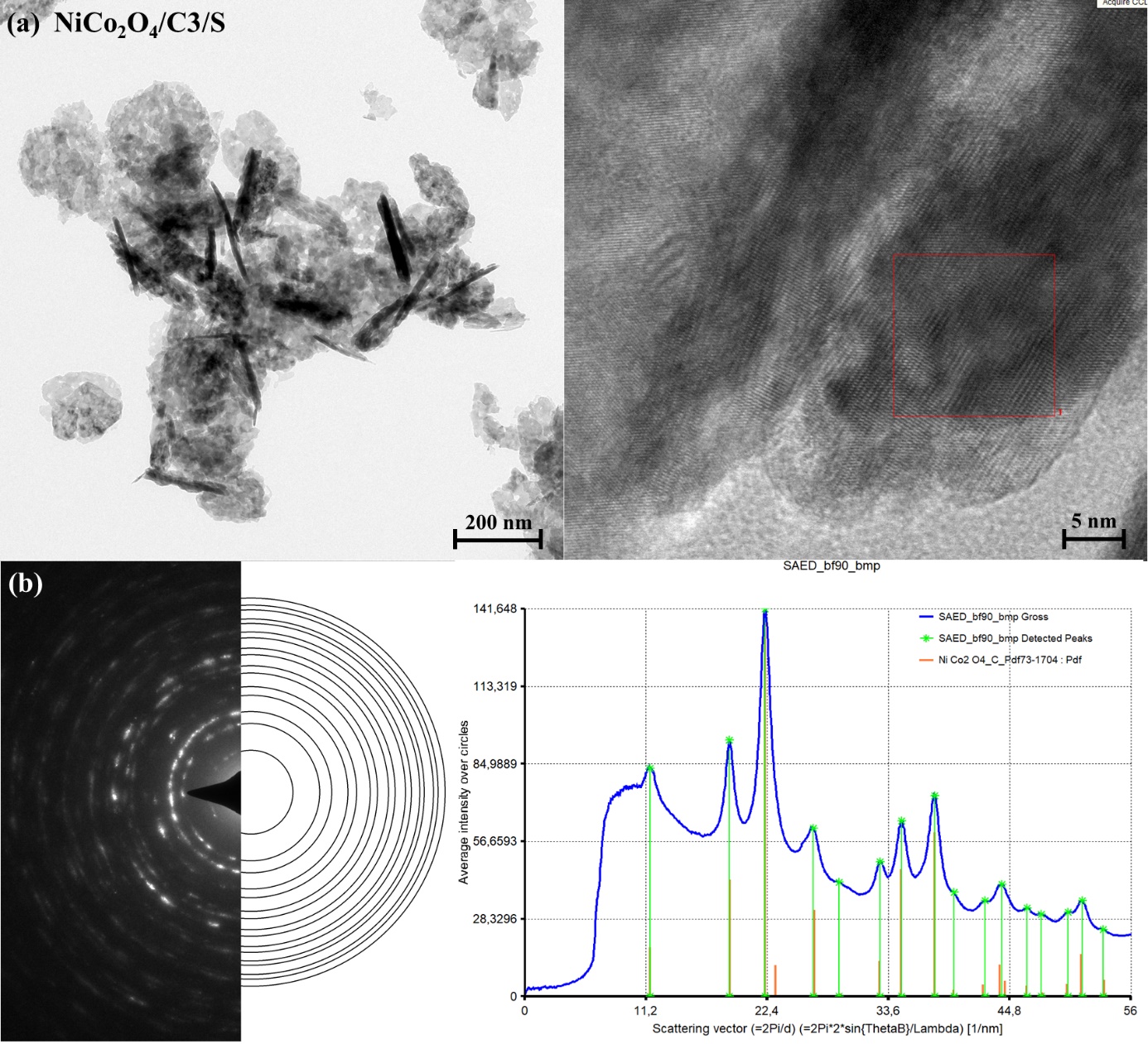


**Figure S5.** (a) TEM images with different magnifications, (b) SAED pattern of NiCo_2_O_4_/C3/S and intensity profile taken from the SAED pattern.


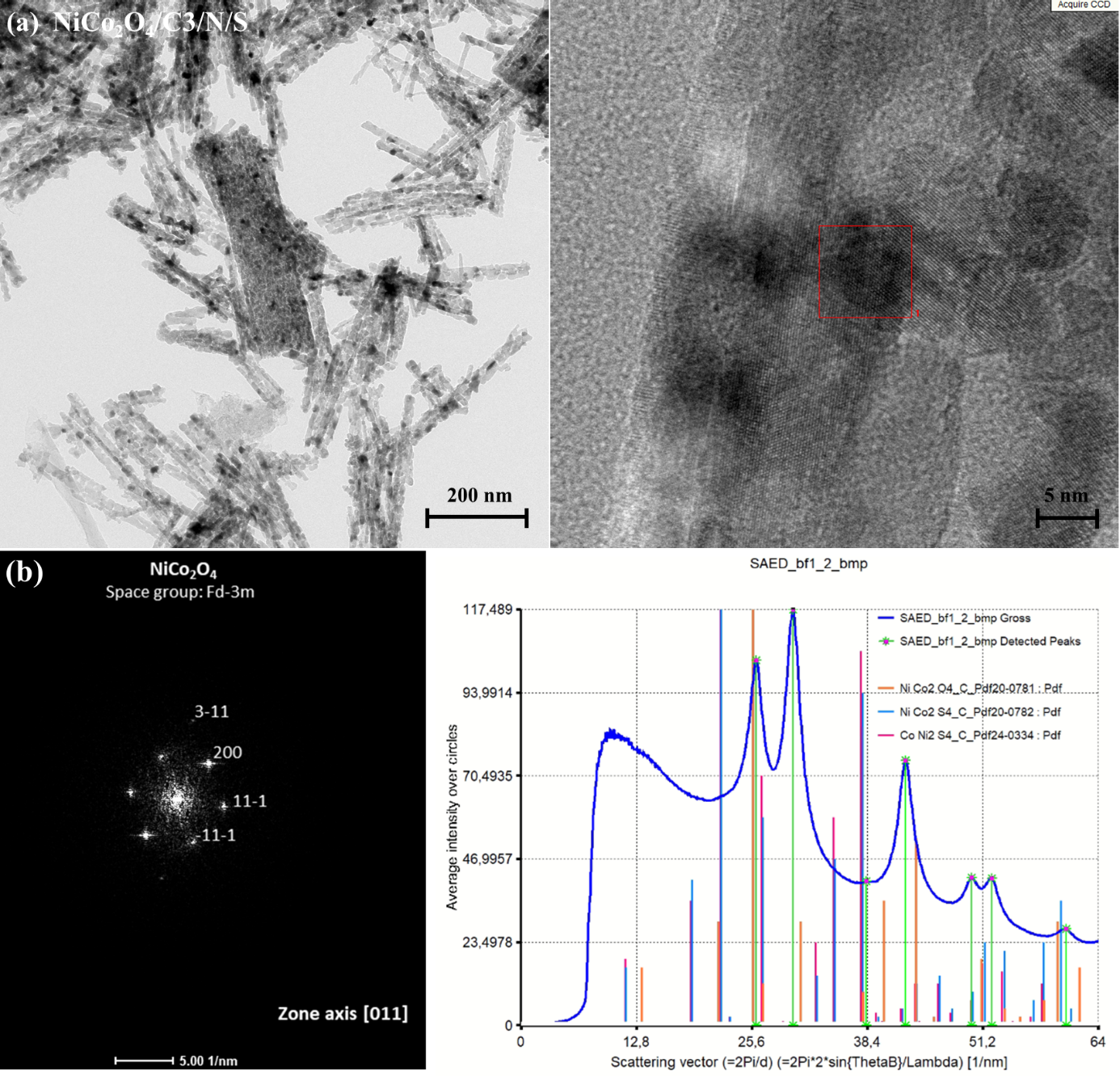


**Figure S6.** (a) TEM images with different magnifications, (b) SAED pattern of NiCo_2_O_4_/C3/N/S and intensity profile taken from the SAED pattern.


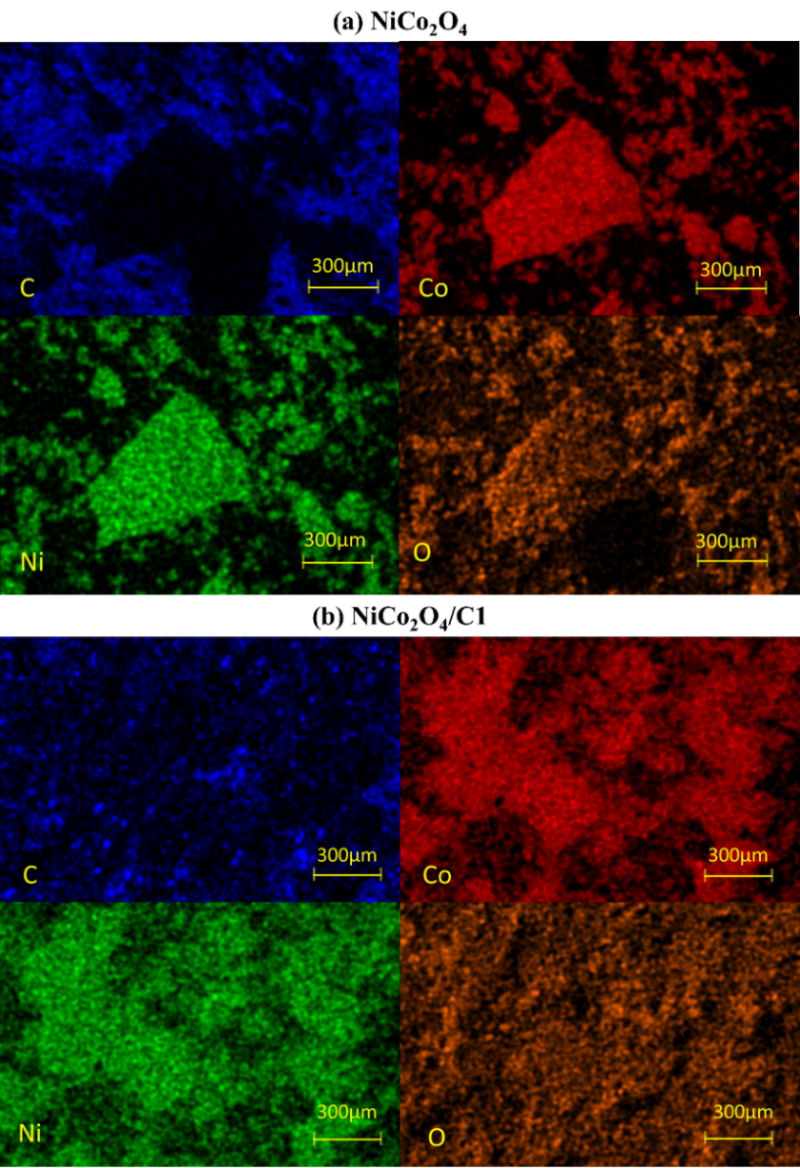


**Figure S7.** Maps of individual elements for the (a) NiCo_2_O_4_ and (b) NiCo_2_O_4_/C1 sample obtained using an EDX spectrometer.


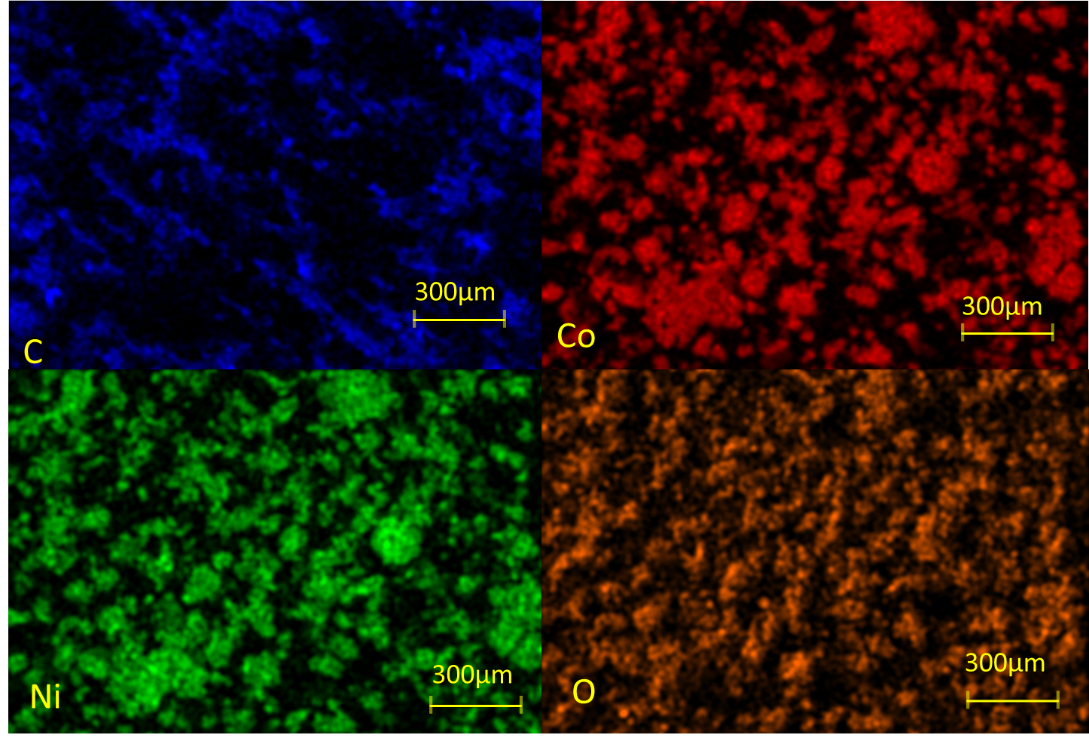


**Figure S8.** Maps of individual elements for the NiCo_2_O_4_/C2 sample obtained using an EDX spectrometer.


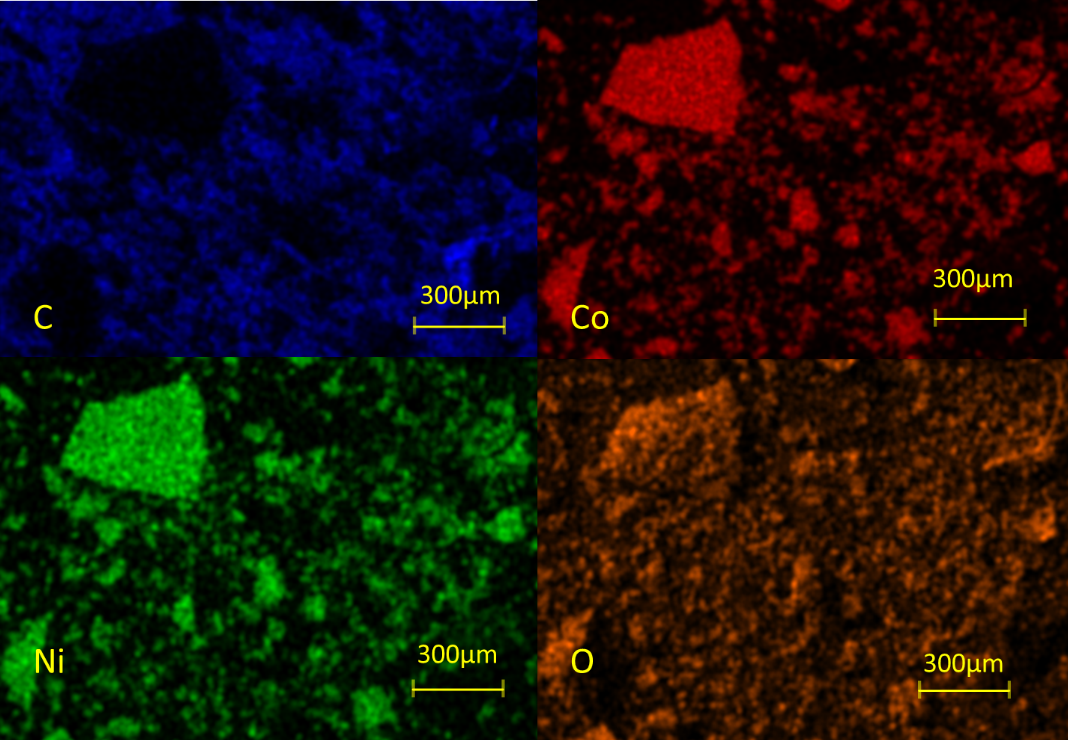


**Figure S9.** Maps of individual elements for the NiCo_2_O_4_/C3 sample obtained using an EDX spectrometer.


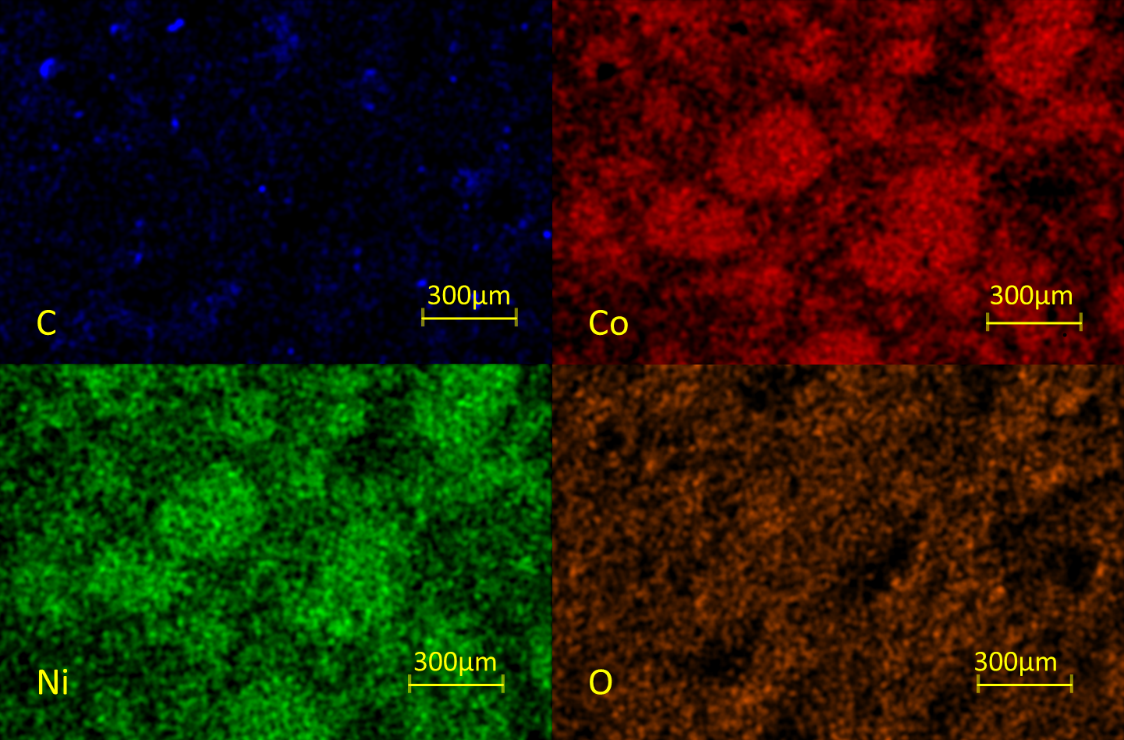


**Figure S10.** Maps of individual elements for the NiCo_2_O_4_/C3/N/S sample obtained using an EDX spectrometer.


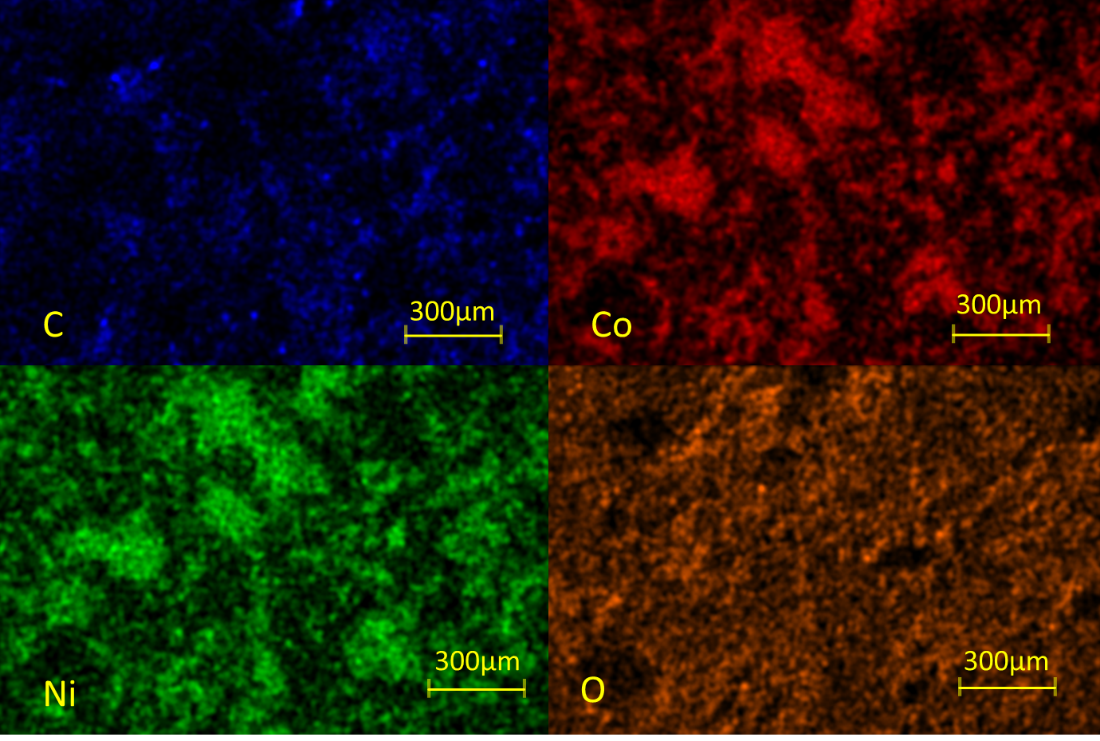


**Figure S11.** Maps of individual elements for the NiCo_2_O_4_/C3/S sample obtained using an EDX spectrometer.

**Table S1.** Elemental composition of the tested catalysts obtained using the EDX detector.

| **Sample** | **Average concentration (% weight)** | | | | | |  |
| --- | --- | --- | --- | --- | --- | --- | --- |
|  | **C** | **O** | **Co** | **Ni** | **N** | **S** | **Al** |
| NiCo_2_O_4_ | 4.65 | 20.19 | 55.74 | 17.13 | ̶ | ̶ | 1.22 |
| NiCo_2_O_4_/C1 | 3.37 | 20.95 | 55.71 | 17.06 | 2.22 | ̶ | 0.69 |
| NiCo_2_O_4_/C2 | 2.82 | 17.22 | 56.79 | 18.29 | 1.76 | ̶ | 2.53 |
| NiCo_2_O_4_/C3 | 1.86 | 18.87 | 55.38 | 17.62 | 2.01 | ̶ | 3.09 |
| NiCo_2_O_4_/C3/N/S | 6.84 | 26.05 | 41.89 | 14.60 | 3.71 | 5.04 | 1.61 |
| NiCo_2_O_4_/C3/S | 3.84 | 26.86 | 47.04 | 17.07 | 2.67 | 0.17 | 1.90 |


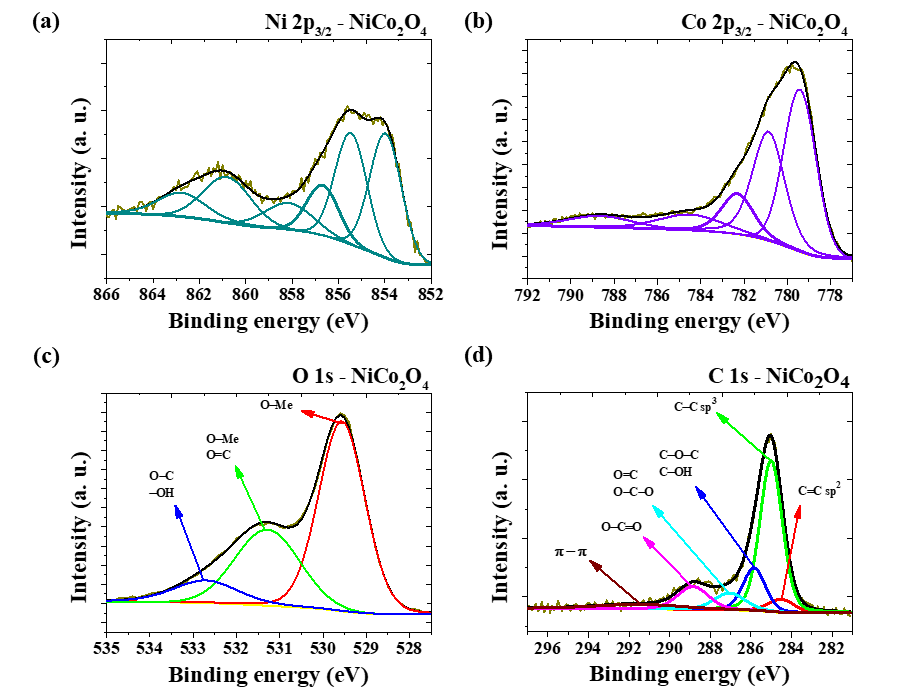


**Figure S12.** High-resolution XPS spectra of NiCo_2_O_4_ sample.


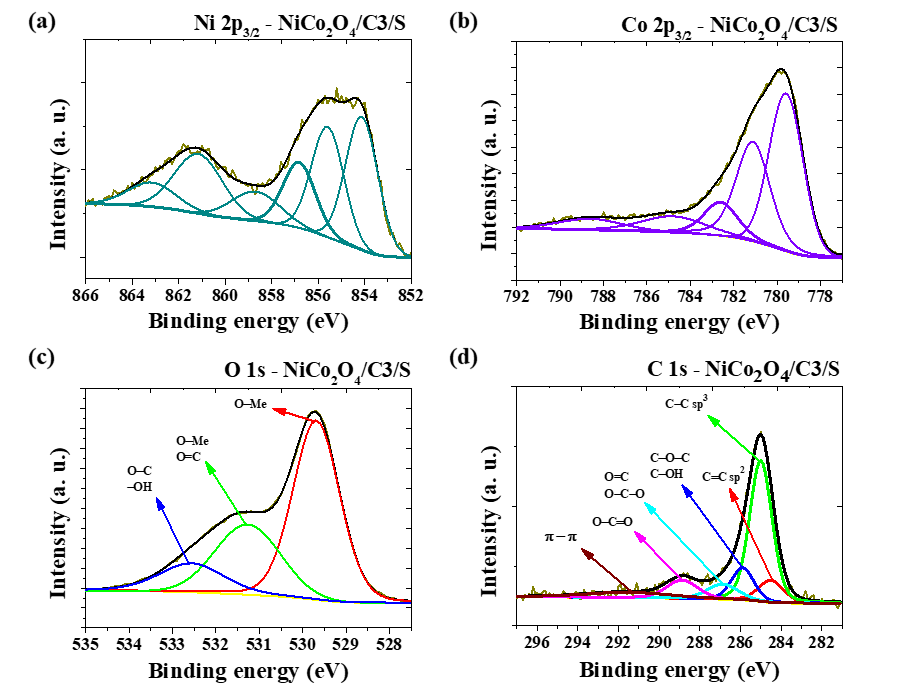


**Figure S13.** High-resolution XPS spectra of NiCo_2_O_4_/C3/S sample.

**Table S2.** Comparison of overpotential values for platinum-based catalysts from literature.

| **Electrocatalyst** | **Overpotential (mV)**  **for *j*_HER_ = 10 mA cm^−2^** | **Reference** |
| --- | --- | --- |
| Pt–Ni nano-multipods | 65 | ^1^ |
| Pt_3_Ni/NiS heterostructures | 200 | ^2^ |
| Ni at-PtNi Nanowires | 30 | ^3^ |
| Pt_3_Ni/NiS Nanowires | 30 | ^4^ |
| Pt/M-AC | 36.1 | ^5^ |
| Nitrogen Doped Reduced Graphene Oxide Based Pt–TiO_2_ | 300 | ^6^ |
| Pt/TiO_2_/Ni(OH)_2_/NF | 96 | ^7^ |
| Pt-MoS_2_ heterostructure | 67.4 | ^8^ |
| Pt-MoO_3−x_ nanoflakes | 69 | ^9^ |


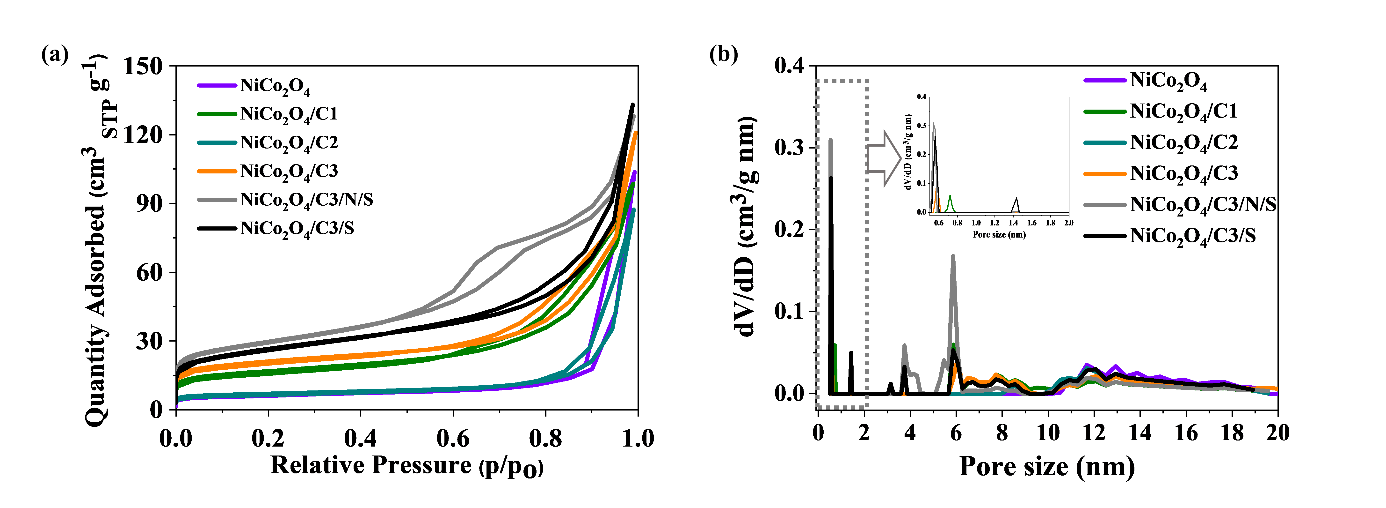


**Figure S14.** (a) N_2_ adsorption-desorption isotherm curves, (b) pore size distribution (PSD) of the synthesized samples.


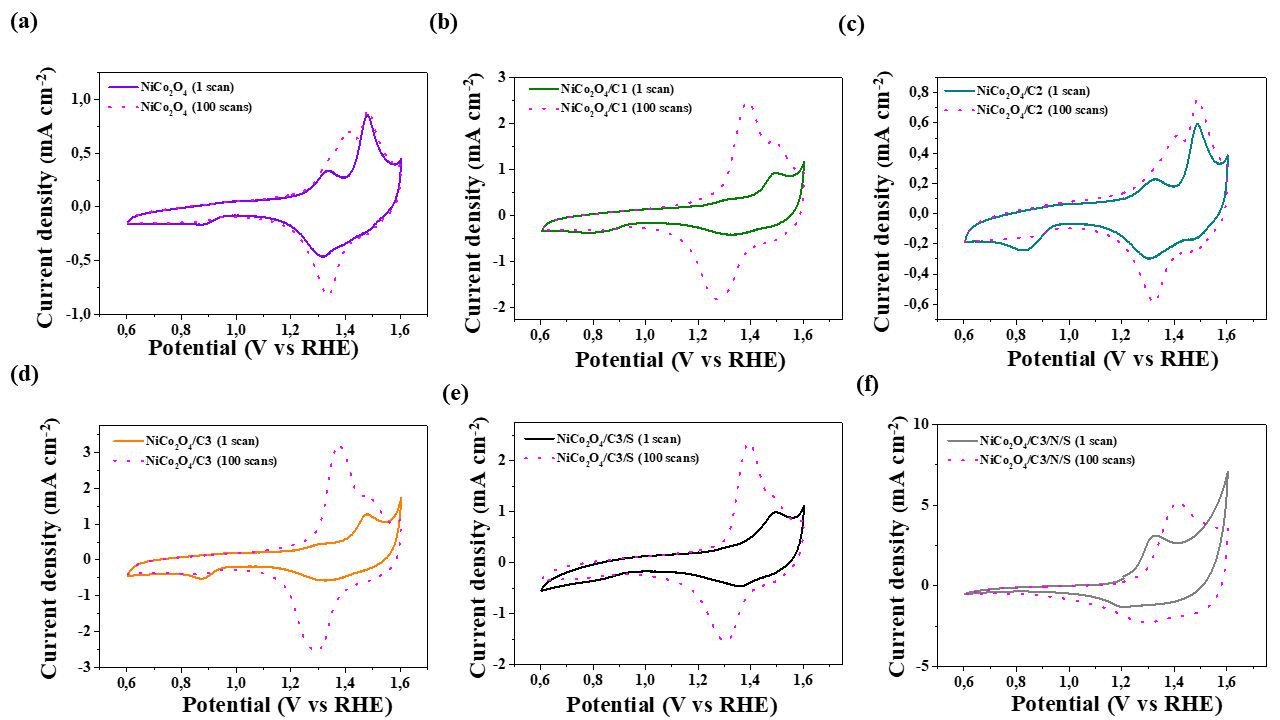


**Figure S15.** Cyclic voltammograms after 1st and 100th cycles for pristine NiCo_2_O_4_ and hybrids with carbon NiCo_2_O_4_/C.


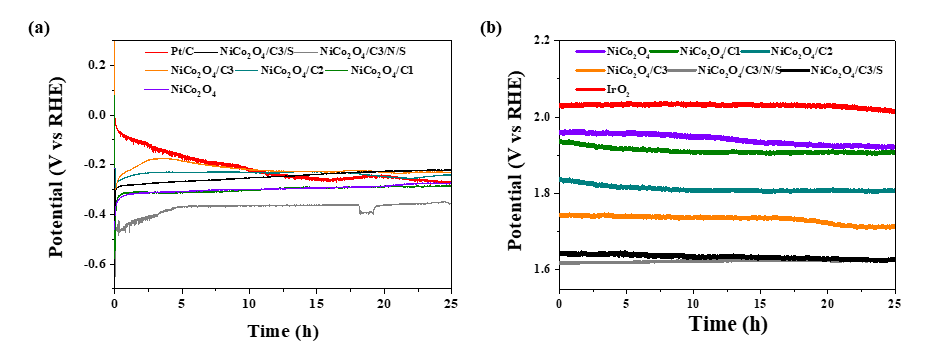


**Figure S16.** Stability of catalysts measured for 25 hours in (a) 0.5 M H_2_SO_4_, (b) 1 M KOH electrolyte solution.

**References:**

1 Cao, Z. *et al.* Platinum-nickel alloy excavated nano-multipods with hexagonal close-packed structure and superior activity towards hydrogen evolution reaction. **8**, 15131 (2017).

2 Miao, J. *et al.* Hierarchical Ni-Mo-S nanosheets on carbon fiber cloth: A flexible electrode for efficient hydrogen generation in neutral electrolyte. **1**, e1500259 (2015).

3 Li, M. *et al.* Single-atom tailoring of platinum nanocatalysts for high-performance multifunctional electrocatalysis. **2**, 495-503 (2019).

4 Wang, P. *et al.* Precise tuning in platinum-nickel/nickel sulfide interface nanowires for synergistic hydrogen evolution catalysis. **8**, 14580 (2017).

5 Chen, H., Luo, X., Huang, S., Yu, F. & Chen, Y. J. J. o. E. C. Phosphorus-doped activated carbon as a platinum-based catalyst support for electrocatalytic hydrogen evolution reaction. **948**, 117820 (2023).

6 Roy, N., Leung, K. T. & Pradhan, D. J. T. J. o. P. C. C. Nitrogen doped reduced graphene oxide based Pt–TiO_2_ nanocomposites for enhanced hydrogen evolution. **119**, 19117-19125 (2015).

7 Kong, A. *et al.* Robust Pt/TiO_2_/Ni(OH)_2_ nanosheet arrays enable outstanding performance for high current density alkaline water electrolysis. **316**, 121654 (2022).

8 Shan, A. *et al.* Interfacial electronic structure modulation of Pt-MoS_2_ heterostructure for enhancing electrocatalytic hydrogen evolution reaction. **94**, 106913 (2022).

9 Lee, D. *et al.* In situ electrochemically synthesized Pt-MoO_3−x_ nanostructure catalysts for efficient hydrogen evolution reaction. **381**, 1-13 (2020).
